# Positive Selection during the Evolution of the Blood Coagulation Factors in the Context of Their Disease-Causing Mutations

**DOI:** 10.1093/molbev/msu248

**Published:** 2014-08-25

**Authors:** Pavithra M. Rallapalli, Christine A. Orengo, Romain A. Studer, Stephen J. Perkins

**Affiliations:** ^1^Department of Structural and Molecular Biology, University College London, London, United Kingdom

**Keywords:** positive selection, coagulation, hemostasis, evolution

## Abstract

Blood coagulation occurs through a cascade of enzymes and cofactors that produces a fibrin clot, while otherwise maintaining hemostasis. The 11 human coagulation factors (FG, FII–FXIII) have been identified across all vertebrates, suggesting that they emerged with the first vertebrates around 500 Ma. Human FVIII, FIX, and FXI are associated with thousands of disease-causing mutations. Here, we evaluated the strength of selective pressures on the 14 genes coding for the 11 factors during vertebrate evolution, and compared these with human mutations in FVIII, FIX, and FXI. Positive selection was identified for fibrinogen (FG), FIII, FVIII, FIX, and FX in the mammalian Primates and Laurasiatheria and the Sauropsida (reptiles and birds). This showed that the coagulation system in vertebrates was under strong selective pressures, perhaps to adapt against blood-invading pathogens. The comparison of these results with disease-causing mutations reported in FVIII, FIX, and FXI showed that the number of disease-causing mutations, and the probability of positive selection were inversely related to each other. It was concluded that when a site was under positive selection, it was less likely to be associated with disease-causing mutations. In contrast, sites under negative selection were more likely to be associated with disease-causing mutations and be destabilizing. A residue-by-residue comparison of the FVIII, FIX, and FXI sequence alignments confirmed this. This improved understanding of evolutionary changes in FVIII, FIX, and FXI provided greater insight into disease-causing mutations, and better assessments of the codon sites that may be mutated in applications of gene therapy.

## Introduction

**Fig. 1.**
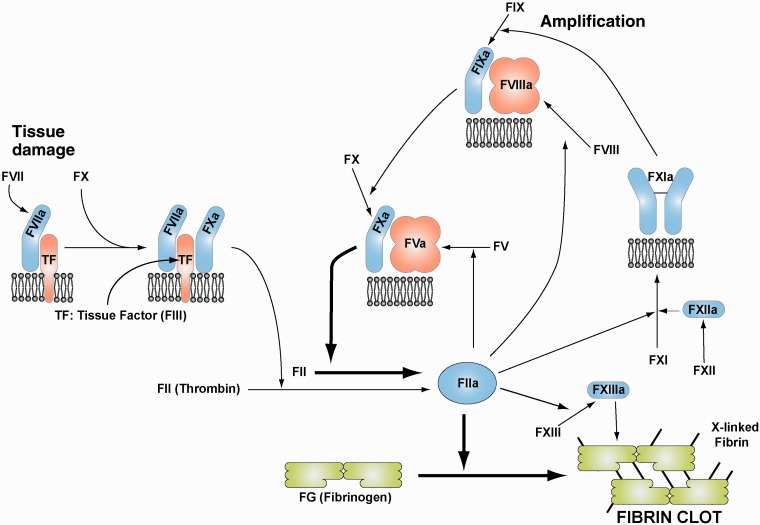
Schema of the blood coagulation pathway leading to fibrin. The relationships between the 11 coagulation factors that are coded by 14 genes are shown in the modern revised coagulation pathway (blue, enzymes; red, cofactors). Tissue factor (TF; also known as FIII) initiates coagulation when it binds and activates FVII. The activated TF-FVIIa complex then activates FX which then generates activated thrombin (FIIa). FVIII, FIX, and FXI enable the amplification of the coagulation pathway to maximize FIIa production, and possess the largest number of pathogenic human mutations. Activated thrombin cleaves FG (FI; green) to form fibrin polymers (blood clots) that are cross-linked by FXIIIa.

Blood coagulation involves a complex yet regulated cascade of over two dozen proteins in blood (Doolittle 2009). Most of these proteins are serine protease enzymes and circulate in blood as inactive zymogens waiting for an activation trigger such as proteolytic cleavage. For coagulation, this trigger is usually some form of vascular injury, followed by activation. In the classical waterfall model, each activated protein goes on to activate the next protein in a rapidly expanding cascade of reactions which quickly results in the local formation of a fibrin clot to seal the injury (Spronk et al. 2003). The 11 human coagulation factor proteins in blood are usually indicated by F and a Roman numeral and followed by a lowercase “a” to indicate their active form, namely FG, FII, FIII, FV, FVII, FVIII, FIX, FX, FXI, FXII, and FXIII (Kawthalkar 2013). FG is comprised of the *α*, *β*, and *γ* genes of fibrinogen (FG) (*FGA*, *FGB*, and *FGG*) while FXIII is produced from two genes *F13A* and *F13B.* Thus these 11 coagulation factor proteins are produced by 14 genes (table 1). The two central processes during coagulation are the conversion of prothrombin (FII) to thrombin (FIIa) that cleaves FG to form fibrin, followed by the polymerization of fibrin to form the fibrin clot. The classic waterfall cascade model involved three pathways (intrinsic, extrinsic, and common) where the intrinsic pathway is first triggered upon injury through FXII and the extrinsic pathway is triggered by the exposure of intracellular tissue factor (FIII) to FVII in serum after which tissue factor binds to and activates FVII. Recent advances in molecular biology have revealed that the waterfall model does not properly account for the roles of tissue factor and FVII (Broze 1995). In the revised waterfall model (fig. 1), thrombin generation occurs in two phases. The initiation phase caused by tissue damage results in relatively low thrombin activation, followed by the amplification (propagation) phase where the bulk of activated thrombin is formed (Butenas et al. 2000). Although the classical model remains useful as a laboratory model of coagulation, the revised model is more effective and logical in laboratory-based screening for coagulation factor abnormalities in bleeding disorders.

**Table 1.** The 14 Coagulation Genes and Their Protein Products.

Gene	ENSEMBL ID	Protein Product	Other Name	Function	Genetic Disorder	Amino Acid Length	No. of Domains	Domain Organization
*FGA*	ENSG00000171560	Coagulation Factor I	Fibrinogen alpha chain	Forms fibrin clot; cofactor in platelet aggregation	Congenital afibrogenmia, familial renal amyloidosis	866	1	Fibrinogen C-terminal domain
*FGB*	ENSG00000171564		Fibrinogen beta chain			491	1	Fibrinogen C-terminal domain
*FGG*	ENSG00000171557		Fibrinogen gamma chain			453	1	FG C-terminal domain
*F2*	ENSG00000180210	Coagulation factor II	Prothrombin	Activates FG to fibrin	Thrombophilia	622	4	Gla–Kringle 1–Kringle 2–SP
*F3*	ENSG00000117525	Coagulation factor III	Tissue factor	Activates FVIIa	—	295	3	Transmembrane–transmembrane helix–transmembrane
*F5*	ENSG00000198734	Coagulation factor V	Proaccelerin	Combines with FX to activate prothrombin	Activated protein C resistance	2224	6	F5/8 type A1–F5/8 type A2–B–F5/8 type A3–F5/8 type C1–F5/8 type C2
*F7*	ENSG00000057593	Coagulation factor FVII	Proconvertin	Activates FIX, FX	Congenital proconvertin/factor VII deficiency	466	4	Gla–EGF1–EGF2–SP
*F8*	ENSG00000185010	Coagulation Factor FVIII	Antihemophilic factor A (AHF-A)	Combines with FIX and FIV to activate FX	Hemophilia A	2351	6	F5/8 type A1–F5/8 type A2–B–F5/8 type A3–F5/8 type C1–F5/8 type C2
*F9*	ENSG00000101981	Coagulation factor FIX	Christmas factor	Combines with FVIII and FIV to activate FX	Hemophilia B	461	4	Gla–EGF1–EGF2–SP
*F10*	ENSG00000126218	Coagulation factor FX	Stuart-prower factor	Converts prothrombin to thrombin	Congenital factor X deficiency	488	4	Gla–EGF1–EGF2–SP
*F11*	ENSG00000088926	Coagulation factor FXI	Plasma thromboplastin antecedent	Combines with FIV to activate FIX	Factor XI deficiency	625	4	Apple 1–apple 2–apple 3–SP
*F12*	ENSG00000131187	Coagulation factor FXII	Hageman factor	Activates FXI; activates plasmin	Hereditary angioedema type III	615	6	Fibronectin type-II–EGF1–fibronectin type-I - EGF2–kringle–SP
*F13A*	ENSG00000124491	Coagulation factor FXIII	Fibrin-stabilizing factor A chain	Stabilizes fibrin	Congenital factor XIIIa deficiency	732	—	—
*F13B*	ENSG00000143278		Fibrin-stabilizing factor B chain	Stabilizes FXIIIA; regulates thrombin	Congenital factor XIIIb deficiency	661	10	Short complement regulator domains 1–10

Many of the coagulation proteins are related to each other via gene duplications that occurred early in vertebrate evolution between the appearance of protochordates and the jawless fish (Doolittle et al. 2008; Doolittle 2009). Two rounds of whole-genome duplications occurred at the beginning of vertebrates, and a third one occurred at the beginning of teleost fishes (Meyer and Van de Peer 2005). In all vertebrates during evolution, blood coagulation retained a central mechanism in which the generation of thrombin resulted in fibrin clot formation. During evolution, several coagulation factors that depend on others for their activity have been altered in a complex fashion, starting from the first vertebrates. Sequence analyses have revealed the order in which the factors evolved (Doolittle 2009). There is considerable interest in the evolutionary development of the complexity of coagulation in mammals. This is driven by the importance of understanding pathogenic disease-causing mutations in humans, as well as understanding how a well-regulated cascade of enzymatic reactions is developed, and obtaining new insights into its molecular mechanism. Analyses of the known mutations in patients and comparison with the mutations tolerated during evolution will clarify which codons are stable and which are not.

The coagulation system overlaps with the innate immune system and the complement proteins through their common properties involving vascular permeability. Deficiencies in the coagulation proteins, mostly due to genetic variations, are associated with a spectrum of genetic disorders that range from life-threatening ones such as severe Hemophilia A (associated with FVIII) to milder variants (table 1). Hemophilia A and B are more common than the others, while some are rare, and these diseases prevail because their underlying genetic mutations are passed on from generation to generation. Replacement therapies using recombinant coagulation proteins are expensive. More recently, FIX gene therapy trials for Hemophilia B patients have been successful (Nathwani et al. 2011), and this method is yet to be applied to FVIII and other coagulation proteins. A major problem faced in FIX gene therapy is the low level of protein expression, in part due to a different codon usage in the organism that produces the protein (Thomas et al. 2003). An evolution-driven study of the codons in the FIX gene may help identify which codons could be altered to increase protein expressions in the producing organism and in the meantime be tolerated in the host.

In order to interpret the effect of evolutionary and mutational changes in coagulation, we have examined FVIII, FIX, and FXI. For FVIII, 5,474 disease-causing mutations have been recently compiled (Rallapalli PM, Kemball-Cook G, Tuddenham EG, Gomez K, Perkins SJ, unpublished data; http://www.factorviii-db.org/, last accessed January 2014), 3,713 mutations were recently published for FIX (Rallapalli et al. 2013; http://www.factorix.org/, last accessed January 2014) and 487 mutations are known for FXI (Saunders et al. 2009; http://www.factorxi.org/, last accessed January 2014). We identified 47 full genomes in the Ensembl database that showed good sequence coverage. This enabled the assessment of positive or negative selection pressures (Yang 2006) in the evolution of coagulation protein sequences. The identification of positive selection in at least nine of the 11 coagulation proteins during different periods of evolution showed that an effective coagulation pathway was under considerable adaptive constraints, even in primates. The mutational analyses for FVIII, FIX, and FXI showed that disease-causing mutations primarily affected highly conserved residues under negative selection, and damaged protein stabilities. This joint evolutionary-mutational study provides novel clarifications of the observed data on pathogenic mutations and may facilitate new gene therapy approaches for their treatments.

## Results

### Selection of 47 Genomes

**Fig. 2.**
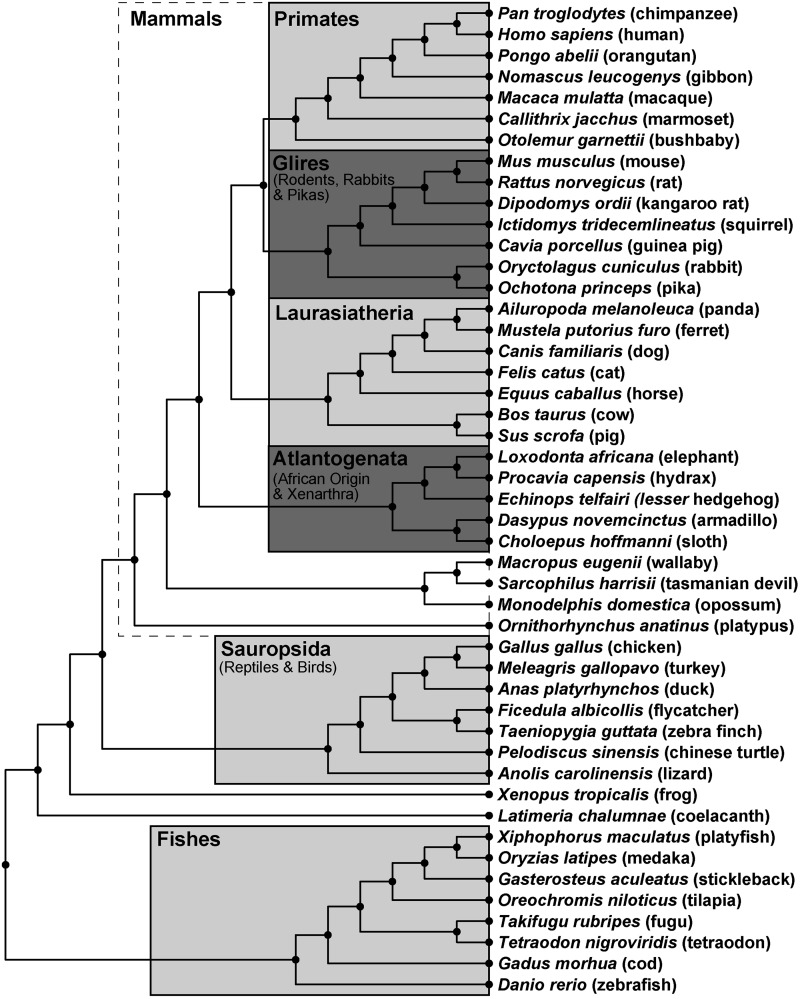
Phylogenetic tree of 47 vertebrate genomes. The tree shows the 47 genomes studied here alongside their clade and taxonomic groups. The 47 vertebrate genomes showed good sequence quality and coverage in the Ensembl database and were grouped into five major clades (Primates, Glires, Laurasiatheria, Sauropsida, and Fishes) and three minor clades. The Mammals comprised of the Primates, Glires, and Laurasiatheria clades together with Atlantogenata (African origin mammals and Xenartha) and four Australian species, totaling 30 out of 47 genomes.

A data set of 14 coagulation factor genes (table 1) that code for 11 proteins across different groups of vertebrate genomes (fig. 2) was identified from the Ensembl database. The Ensembl database currently holds 78 genomes. Of these, only 47 vertebrate genomes were chosen based on their sequence coverage and assembly quality (fig. 2). Ensembl gene sets are built from assemblies of DNA sequences and protein information, hence it was important to select only good-quality assemblies for the gene trees and sequence data sets used in this study. The 47 vertebrate genomes were classified into five major clades based on the Ensembl classification, namely Primates, Glires, Laurasiatheria, Sauropsida, and Fishes. The mammalian clade of 30 genomes consisted of three clades used in our study (Primates, Glires, and Laurasiatheria) and other clades (Atlantogenata and Australian mammals) (fig. 2). The codon substitution site-models M1a, M2a, M7, M8, and M8a from CodeML (Materials and Methods) were used to calculate the selection pressures on the five major clades, each with at least seven sequences, to ensure that the CodeML calculations were reasonably powerful (Anisimova et al. 2002). By following this criterion, the Atlantogenata clade with only five sequences was eliminated from the selection pressure calculations. The multiple sequence alignments from Ensembl were realigned using a strict quality control procedure (supplementary fig. S1, Supplementary Material online), which was performed in order to minimize the number of false positive results below.

### Presence and Absence of Coagulation Factors

The 47 genomes were searched for the 14 genes coding for the 11 coagulation factors. In cases where the Ensembl gene tree for a given coagulation factor did not show the presence of a particular genome, we searched for putative genes across that genomic sequence to establish whether the organism had lost them. The purpose of the coagulation cascade is to convert FG into the fibrin clot, and unsurprisingly FG (the *FGA*, *FGB,* and *FGG* genes) was found in all 47 genomes. The other central genes thrombin (*F2*), tissue factor (*F3*), the coagulation cascade cofactors (*F5* and *F8*), *F7*, *F9,* and *F10* were present across all 47 genomes from fishes to humans. *F11* is present in all Sarcopterygii (tetrapods and coelacanth), but seems to have been lost or highly diverged in teleost fishes, because it is only recorded in the spotted gar *Lepisosteus oculatus*, which belongs to Holostei, a sister clade of teleost fishes. *F12* appeared in the ancestor of tetrapods, and is absent in fishes, but present in amphibians (*Xenopus tropicalis*), reptiles (*Anolis carolinensis*), and mammals. For *F13*, the alpha chain (*F13A*) was observed to appear from the time of vertebrates, because this gene has been found in the sea lamprey, fishes, and tetrapods. The beta chain (*F13B*) appears to be present at the origin of vertebrates, because *F13B* sequences have been recorded in cave fish (*Astyanax mexicanus*) and medaka (*Oryzias latipes*). Occasionally a gene was not reported; this was most likely to arise from incomplete or unidentified sequences.

### Selective Pressures Using Codon Substitution Site-Models

Codon substitution site-models from the CodeML package were used to analyze the protein-coding sequences in the 14 genes following the quality control of their alignments. The models assume that selective pressure does not act equally on the entire protein sequence but varies between amino acid sites in a gene family. We calculated the log likelihood values (lnL) of five different selection models (M1a, M2a, M7, M8, and M8a) in order to perform the likelihood ratio test (LRT). The five models differed from each other in the number of parameters (np) considered and their variable *ω* values. The *ω* value is the ratio of nonsynonymous and synonymous substitution rates (*d*_N_/*d*_S_) (Materials and Methods). The comparison between a null model that does not consider *ω* > 1 (M1a, M7, or M8a) and an alternate model that considers *ω* > 1 (M2a or M8) is a measure of positive selection. If the null (neutral) model is rejected in favor of the alternate (selection) model, positive selection is inferred (Yang 2006).

To evaluate the five models, the LRT value was calculated as follows:

$$\text{LRT }=2({\text{lnL}}_{\text{alternate }}-{\text{lnL}}_{\text{null}})$$

where lnL is the maximum-likelihood estimate of the probabilities of selection. The LRT is asymptotically distributed as a ${\text{χ}}_{k}^{2}$ function where *k* (also known as the degrees of freedom) is the difference between the np in the null and alternate models. The np values were calculated along with the lnL values in CodeML.

$$k={\text{np}}_{\text{alternate}}-{\text{ np}}_{\text{null}}$$

The LRT values for which the ${\text{χ}}_{k}^{2}$ distribution shows a *P* value < 0.05 indicated that some sites were significantly under positive selection. For this study, the comparison of the M8a null model with the M8 alternate model (referred to as M8a-M8) was considered optimal to infer positive selection (table 2). The LRT M1a-M2a is too conservative, whereas M7–M8 was considered to be more problem prone and less accurate compared with M8a-M8 (Wong et al. 2004; Studer and Robinson-Rechavi 2009b). Because the *P* values result from multiple tests performed using different genes at different clade levels, it was necessary to control for the expected proportion of false positives. False positives were accounted for by calculating the *q* value correction over the *P* value for the different genes in each clade, this approach being both powerful and specific (Studer et al. 2008).

**Table 2.** LRT Statistics for the Site Model Comparisons of Model 8 with Model 8a for the 14 Coagulation Factors among the Five Clades.

Gene	Clade	No. of Sequences	No. of Codons	(M8a-M8)		
				LRT	*P* value	q value
*FGA*	Vertebrates	44	236	1.39	0.24	0.35
	Mammals	29	288	7.75	0.01**	0.04*
	Primates	7	865	3.35	0.07	0.16
	Glires	7	670	2.89	0.09	0.19
	Laurasiatheria	9	267	7.28	0.01**	0.04*
	Sauropsida	6	464	0.33	0.57	0.62
	Fishes	7	592	−0.16	1.00	0.72
*FGB*	Vertebrates	40	429	1.45	0.23	0.35
	Mammals	26	456	−2.78	1.00	0.72
	Primates	6	488	7.44	0.01**	0.04*
	Glires	6	471	0.69	0.41	0.51
	Laurasiatheria	8	479	1.44	0.23	0.35
	Sauropsida	5	473	−0.02	1.00	0.72
	Fishes	7	476	−0.99	1.00	0.72
*FGG*	Vertebrates	44	386	−1.74	1.00	0.72
	Mammals	29	427	3.19	0.07	0.17
	Primates	7	433	3.98	0.05*	0.13
	Glires	7	433	3.44	0.06	0.16
	Laurasiatheria	8	432	0.09	0.76	0.72
	Sauropsida	5	427	0.03	0.86	0.72
	Fishes	8	395	−5.42	1.00	0.72
*F2*	Vertebrates	38	475	16.91	0.00**	0.00**
	Mammals	23	571	4.64	0.03*	0.11
	Primates	7	621	4.25	0.04*	0.12
	Glires	5	611	−0.07	1.00	0.72
	Laurasiatheria	7	620	1.58	0.21	0.34
	Sauropsida	5	593	3.68	0.05	0.14
	Fishes	8	562	−0.05	1.00	0.72
*F3*	Vertebrates	47	166	2.09	0.15	0.27
	Mammals	26	240	13.37	0.00**	0.00**
	Primates	7	295	0.01	0.90	0.72
	Glires	6	287	3.07	0.08	0.18
	Laurasiatheria	7	292	0.97	0.32	0.44
	**Sauropsida**	5	254	8.65	0.00**	0.02*
	Fishes	14	192	0.82	0.36	0.47
*F5*	**Vertebrates**	37	1,182	14.95	0.00**	0.00**
	**Mammals**	22	1,741	31.92	0.00**	0.00**
	**Primates**	6	2,116	5.36	0.02*	0.08*
	Glires	6	1,847	0.48	0.49	0.56
	Laurasiatheria	6	2,043	4.83	0.03*	0.10
	Sauropsida	5	1,550	1.45	0.23	0.35
	Fishes	8	1,534	2.12	0.15	0.27
*F7*	**Vertebrates**	34	330	5.93	0.01**	0.07*
	Mammals	29	344	3.06	0.08	0.18
	Primates	7	443	0.16	0.69	0.72
	Glires	7	417	−0.3	1.00	0.72
	Laurasiatheria	7	443	0.57	0.45	0.54
	Sauropsida	3	425	0.55	0.46	0.54
	Fishes	**NA**				
*F8*	Vertebrates	38	1,028	4.85	0.03*	0.10
	**Mammals**	24	1,985	11.19	0.00**	0.01**
	**Primates**	7	2,351	6.83	0.01**	0.04*
	Glires	5	2,257	3.95	0.05*	0.13
	Laurasiatheria	7	2,344	0.25	0.62	0.67
	Sauropsida	5	1,480	0.03	0.86	0.72
	Fishes	7	1,413	0.9	0.34	0.45
*F9*	Vertebrates	44	329	0.63	0.43	0.52
	**Mammals**	24	443	18.57	0.00**	0.00**
	Primates	7	461	1.28	0.26	0.36
	Glires	5	468	1.85	0.17	0.30
	Laurasiatheria	7	459	4.34	0.04*	0.12
	Sauropsida	5	435	−0.03	1.00	0.72
	Fishes	13	368	−0.36	1.00	0.72
*F10*	**Vertebrates**	41	353	14.55	0.00**	0.00**
	Mammals	26	424	−0.23	0.63	0.67
	Primates	7	485	0.01	0.91	0.72
	Glires	6	464	2.06	0.15	0.27
	Laurasiatheria	7	463	2.3	0.13	0.25
	Sauropsida	5	385	0.03	0.87	0.72
	Fishes	8	391	0.71	0.40	0.51
*F11*	Vertebrates	23	614	0.34	0.56	0.62
	Mammals	23	614	0.34	0.56	0.62
	Primates	7	625	0.05	0.82	0.72
	Glires	5	619	0.07	0.79	0.72
	Laurasiatheria	6	625	0.02	0.89	0.72
	Sauropsida	**NA**				
	Fishes	**NA**				
*F12*	Vertebrates	27	512	−3.98	1.00	0.72
	Mammals	25	533	−0.18	1.00	0.72
	Primates	7	615	−0.02	1.00	0.72
	Glires	6	557	−0.07	1.00	0.72
	Laurasiatheria	8	513	4.6	0.03*	0.11
	Sauropsida	**NA**				
	Fishes	**NA**				
*F13A*	**Vertebrates**	47	631	9.07	0.00**	0.02*
	Mammals	26	684	2.58	0.11	0.22
	Primates	7	732	1.62	0.20	0.34
	Glires	6	730	−1.47	1.00	0.72
	Laurasiatheria	9	730	−0.27	1.00	0.72
	Sauropsida	5	732	0.01	0.92	0.72
	Fishes	14	643	2.59	0.11	0.22
*F13B*	Vertebrates	31	609	1.3	0.25	0.36
	Mammals	25	640	1.57	0.21	0.34
	Primates	7	660	1.22	0.27	0.37
	Glires	5	634	−0.21	1.00	0.72
	**Laurasiatheria**	7	656	7.08	0.01**	0.04*
	Sauropsida	5	573	−0.93	1.00	0.72
	Fishes	**NA**				

NOTE.—Positive selection is denoted in bold in column 2.

*P < 0.05; **P < 0.01

**Fig. 3.**
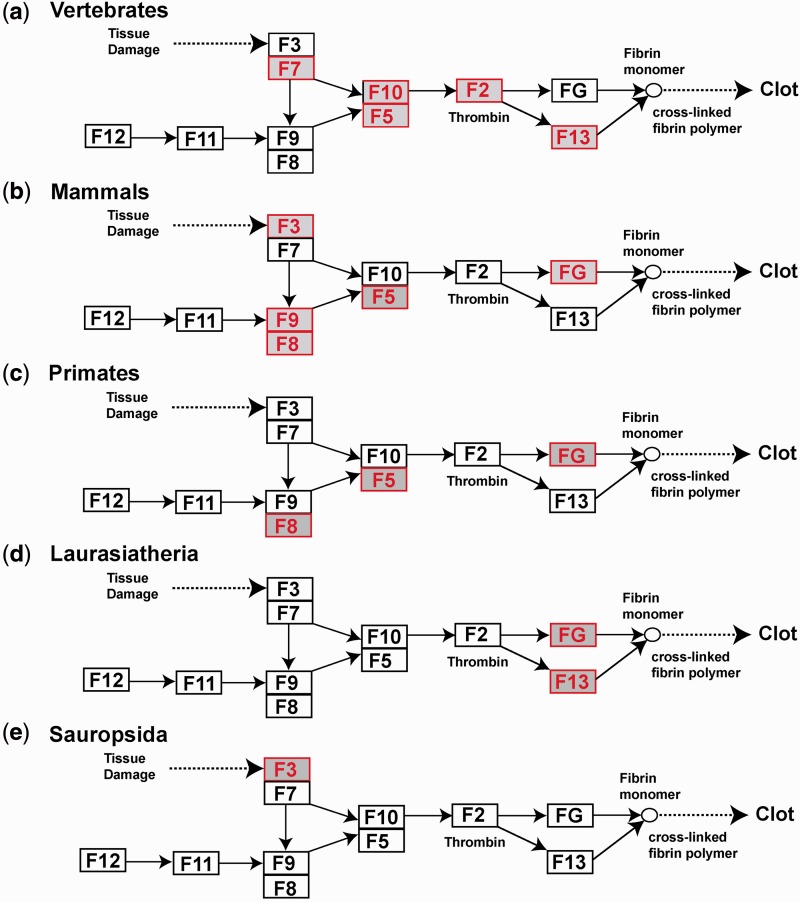
Positively selected genes of the coagulation cascade in vertebrates, mammals, and three major clades. The layout of this figure is a simplified representation of figure 1. Genes showing significant positive selection in the LRT calculations are highlighted in red. (*a*) Positive selection was observed in five genes when all 47 genome sequences (vertebrates) were analyzed as a single group. (*b*) In the Mammals group (fig. 2), where positive selection is seen for five genes, only the (*c*) Primates and (*d*) Laurasiatheria clades are shown with positive selection for two or three genes only, because no positive selection was seen in the Glires clade whereas insufficient sequences were available in the Atlantogenata clade for conclusions to be drawn. (*e*) Positive selection was seen in the Sauropsida, clade but no positive selection was observed in Fishes (fig. 2).

The overall picture is that a total of nine coagulation factor proteins (out of the 11 of the cascade) underwent positive selection at one or more stages in evolution (fig. 3). The highest number of genes under positive selection within an individual clade is five (for vertebrates and mammals). For Vertebrates, using the LRT M8a-M8, positive selection was found in as many as five of the 14 coagulation genes (table 2). In figure 3*a*, these included *F2*, *F5*, *F7*, *F10,* and *F13A* for all 47 vertebrates. In figure 3*b*, these included *FGA*, *F3*, *F5*, *F8,* and *F9* for the 30 mammals. In figure 3*c*–*e*, these included *FGB*, *F5,* and *F8* (Primates); *FGA* and *F13B* (Laurasiatheria), and *F3* (Sauropsida). *FG* (*FG = FGA + FGB + FGG*) and *F5* were observed to undergo the most extensive positive selection compared with any other coagulation genes. The selective pressure on all the genes in different clades was identified from the LRT values (table 2), except for those of *F11* in Laurasiatheria, Sauropsida, and Fishes, *F12* in Sauropsida and Fishes, and *F13B* in Fishes because these genes were absent (supplementary table S1, Supplementary Material online).

### Estimation of Selective Pressures Using Branch-Site Model of Codon Substitution

The above site models estimated selective pressures that vary among amino acid positions across multiple species. But selective pressures can also vary among species (Studer and Robinson-Rechavi 2009a), and codon substitution branch-site models have been developed to detect such episodic positive selection (Zhang et al. 2005). Here, we have used the results of the branch-site model from two different sources, one from the Selectome database, and the other from our own calculations. Both approaches employed CodeML on the two data sets of this study (Materials and Methods) in order to check for consistency with the positive selection identified in the site-model calculations (fig. 3). Similar to the site model, the false positives in our branch-site model calculations were accounted for by calculating the *q* value correction over the *P* value for the different genes in each clade. For both branch-site calculations, we followed the taxonomic grouping in the Selectome database (supplementary table S2*b*, Supplementary Material online). The highest order of classification is the Sarcopterygii (coelacanths, lungfish, and tetrapods), followed by its largest subgroup Tetrapoda (mammals, sauropsids, and frogs). Other major taxonomic subtrees were those of amninots, sauropsids, mammals, theria, and eutheria. The Actinopterygii (fishes) and Sauropsida (birds) are common to both the calculations. The Eutheria and mammalian subtree calculations from the Selectome database were considered to be equivalent to the mammalian clade of our analyses (fig. 2). Positive selection across the vertebrates was observed for all genes except *FGG, F3, F7,* and *F11* in our branch-site model calculations (supplementary table S2*b*, Supplementary Material online). We obtained similar results with our site-model calculations (supplementary table S2*a*, Supplementary Material online), except for the exclusion of *F3* and *F7* (fig. 3*a*). Our branch-site calculations identified positive selection in mammalian *FGB*, *F2*, *F8,* and *F9*, whereas the site model identified positive selection in mammalian *FGA*, *F3*, *F5*, *F8,* and *F9*. Overall *FG (FGA, FGB), F8* and *F9* were consistently identified to be positively selected in mammals, whereas *F5* shows positive selection up to amniotes, after which the mammals and the sauropsids clade split. In conclusion, the two branch-site and the site models all suggested signatures of positive selections across *F8* and *F9*, whereas no such selection was observed across and between *F11*.

### Identification of Codon Sites under Positive Selection

For up to 11 genes out of 14 identified to be under positive selection (table 3), further analyses were performed to identify which codon sites were under such selection. This was done using the Bayes Empirical Bayes (BEB) method from the M8 site-model (Yang et al. 2005). The number of codons potentially under positive selection (BEB >50%) in the positively selected genes ranged between 3 and 64. But the number of codons accurately predicted under positive selection (BEB >95%) is actually much lower (between 0 and 4). The *FGA* gene in Laurasiatheria showed the highest percentage of positively selected codons (13.29%, as estimated by CodeML, and 64 codons detect, with two at BEB >95%), followed by the *F5* gene in Primates (13.21%, with 54 codons, all BEB <95 %). The smallest percentage of positively selected codons was observed in the *F10* gene of Vertebrates (0.01% with three codons, all with BEB <95%). No positive selection was observed in any of the codon sites for the *FGG*, *F11*, and *F12* genes in the taxa groups of this study.

**Table 3.** Positively Selected Sites in the M8a-M8 Comparison.

Gene	Clade	No. of Codons Analyzed	No. of Codons under positive selection	Percentage Estimated by CodeML (%)	Sites under Positive Selection (BEB>50%)
*FGA*	Mammals	288	13	7.17	7,18,32,35,39,51,101,108,178*,212,219,246,273
*FGA*	Laurasiatheria	267	64	13.29	4,6*,7,12,19,29,30,33,36,40,52,93,94,96,97,100,102,103,108,109,116,117,118,123,136,138,141,143,155,171,180,182,195,208,210,213,214,216,218,219,220,222,223,225,227,229,231,232,233,234,236,239,242,244,245,250,253,254*,256,259, 260,261,264,265
*FGB*	Primates	488	17	8.22	39*,41,55,80,81,82,130,134,135*,137,173,290, 367,388,413,415,448
*F2*	Vertebrates	475	5	0.99	52,57,151,162,381
*F3*	Mammals	240	11	6.63	55,72,76,91,115,177,179,185*,195,230,240
*F3*	Sauropsida	254	13	10.60	3,40,41,44,74,79,80,81,110,123,170,208,249*
*F5*	Vertebrates	1,182	10	0.87	103,275,574**,578*,593*,602,777,926,995,1010
*F5*	Mammals	1,741	54	2.72	2,3,7,24,36,60,134**,389*,392,656,663,667,669, 676**,680*,718,720,731,745,749,765,768,782,809,819,822,824,875,880,894,895,907,911,916,928,930,945,967,976,989,1016,1040,1076,1080,1089,1136,1139,1324,1471,1479,1515,1522,1548,1564
*F5*	Primates	2,116	54	13.21	7,40,52,129,180,211,336,341,408,409,434, 592,660,703,707,720,754,784,877,879,888,892,907,941,959,978,1033,1039,1059,1131,1168,1205,1209,1218,1220,1222,1230,1231,1232,1236,1244,1254,1262,1265,1279,1285,1498,1504,1519,1526,1667,1682,1849,1918
*F7*	Vertebrates	330	12	5.22	5,6*,52,66,88*,162,167,178,179,209,258,262
*F8*	Mammals	1,985	27	3.98	7,245,281,339,342,521,667,718,765,784, 785,792,807,817,889,912,1130,1193,1197, 1220,1229,1288,1308,1333,1686,1967,1984
*F8*	Primates	2,351	33	0.63	422,755,791,814,850,860,886,927,964,966, 979,988,996,1019,1071,1180,1282,1312, 1361,1414,1421,1436,1438,1459,1527,1582, 1613,1622,1650,1689,1730,2340,2349
*F9*	Mammals	443	15	4.40	4,5,100*,114**,140*,150,177,181, 199**,202,206,209,270,309,348
*F10*	Vertebrates	353	3	0.01	1,44,70
*F13A*	Vertebrates	631	6	1.26	398,489*,497,502,550,613
*F13B*	Laurasiatheria	656	18	1.52	5,56,73,78,107,341,349,350,374,443, 459,461,497,522,525,539,540,624

*P > 95%; **P > 99%.

### Disease-Causing Missense Mutations and Relationship to Evolutionary Pressures

The relationship between selective pressure and the probability of disease-causing mutations at a codon site is of great interest. Disease-causing missense mutations have been reported in all 14 coagulation genes, in particular for Hemophilia A (*F8*), Hemophilia B (*F9*), factor XI deficiency (*F11*), and thrombophilia (*F2*) (table 1). Extensive compilations of 487–5,474 disease-causing mutations are available for three of these coagulation factors FVIII, FIX, and FXI*,* (Saunders et al. 2009; Rallapalli et al. 2013; Rallapalli et al. 2014 [unpublished data]). There is a sufficient number of pathogenic mutation and evolutionary selection data from the FVIII, FIX, and FXI proteins to enable us to compare in statistical detail the correlation between selective pressure and disease-causing mutations.

**Fig. 4.**
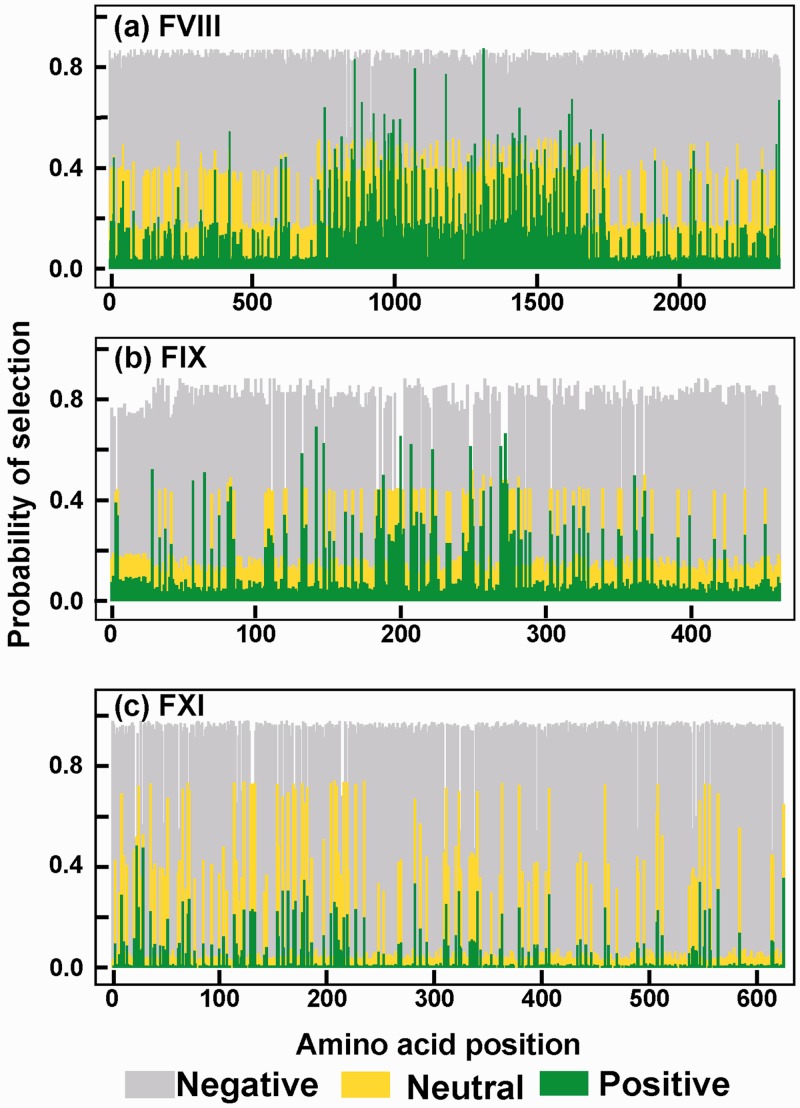
Probability of evolutionary selection across the Primates clade for the three proteins FVIII, FIX, and FXI. The probabilities of negative, neutral, and positive selection were plotted against the amino acid position in the three proteins. The probability values were obtained from CodeML analyses of the seven primate sequences (gray, negative selection; yellow, neutral selection; green, positive selection).

For the comparison of the FVIII, FIX, and FXI protein changes, selective pressures were identified using the BEB inference method from model M2a (fig. 4). While powerful, a disadvantage of Model M8 is that it classifies sites into 11 categories (ten in negative/neutral and one positive selection). These 11 categories can vary between different gene families, making the comparison difficult. Thus, model M2a is advantageous in that this has only three distinct categories, namely negative selection (*d*_N_/*d*_S_ < 1), neutral evolution (*d*_N_/*d*_S_ = 1) and positive selection (*d*_N_/*d*_S_ > 1). Under the M1a-M2a test in the primates clade, two genes (*F8* and *F9*) showed positive selection, whereas *F11* did not show positive selection, which differs from that shown in figure 3*c* which is based on the M8 model. The posterior probabilities of neutral, negative, and positive selection for the FVIII, FIX, and FXI proteins showed that positive selection occurred in specific codons, the most being for *F8* and the fewest for *F11*. For completion, the same analysis for the other 11 genes is shown in supplementary table S2, Supplementary Material online. These show similar outcomes to those for FVIII, FIX, and FXI.

**Fig. 5.**
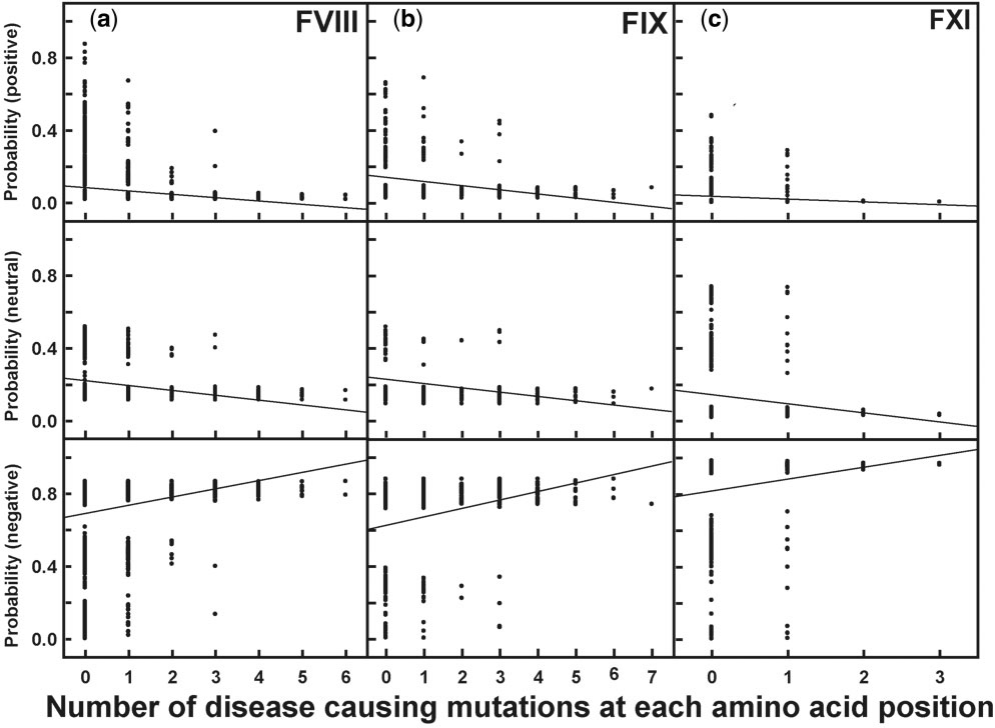
Relationship between disease-causing mutations and evolutionary pressures. The correlation between evolutionary selection and the number of times a disease-causing mutation occurs at each amino acid position is shown. In the columns, (*a*) FVIII, (*b*) FIX, and (*c*) FXI correspond to the three coagulation factors for which pathogenic mutational information is available in sufficient quantity from our three mutational databases. The three rows correspond to the posterior probability (based on the BEB model of CodeML) of positive selection, neutral evolution, and negative selection for primates.

For each amino acid position in FVIII, FIX, and FXI, we compared its selective pressure (positive, neutral, or negative) with the reported number of disease-causing mutations. The outcome was presented using regression plots (fig. 5). The regression plots and their correlation coefficients revealed the relationship between the selection probability values at amino acid sites with the disease-causing mutations at those sites. The average probability of a site to be under positive selection and its number of disease-causing mutations showed a negative relationship with correlation coefficients of −0.85, −0.67, and −0.95 in FVIII, FIX, and FXI, respectively (fig. 5, top row). The correlation coefficients for neutral selection followed that of positive selection with values of −0.87, −0.64, and −0.96 for FVIII, FIX, and FXI, respectively (fig. 5, middle row). In distinction to these, a positive relationship was seen between the average probability of negative selection and the disease-causing mutations, with correlation coefficients of +0.86, +0.66, and +0.95 for FVIII, FIX, and FXI, respectively (fig. 5, bottom row). To summarize, the fewer the number of disease mutations at a given site, the higher is the probability for positive selection or neutral evolution (fig. 5, top row and middle row). The greater the number of disease-causing mutations at a given site, the higher the probability for negative selection (fig. 5, bottom row). The comparison between the positively selected proteins FVIII and FIX with the nonpositively selected protein FXI is noteworthy. Irrespective of the selective pressure, all three proteins exhibited similar regression lines in each of the three rows. These comparisons show that there is a general inverse proportionality relationship between the probability of positive selection (or neutral evolution) and the number of disease-causing mutations at each site. This confirms previous observations that disease-causing mutations tend to occur at critical sites in the protein and that these highly conserved sites are less tolerant to new mutations (negative selection).

### Stability Effect of Mutations

**Fig. 6.**
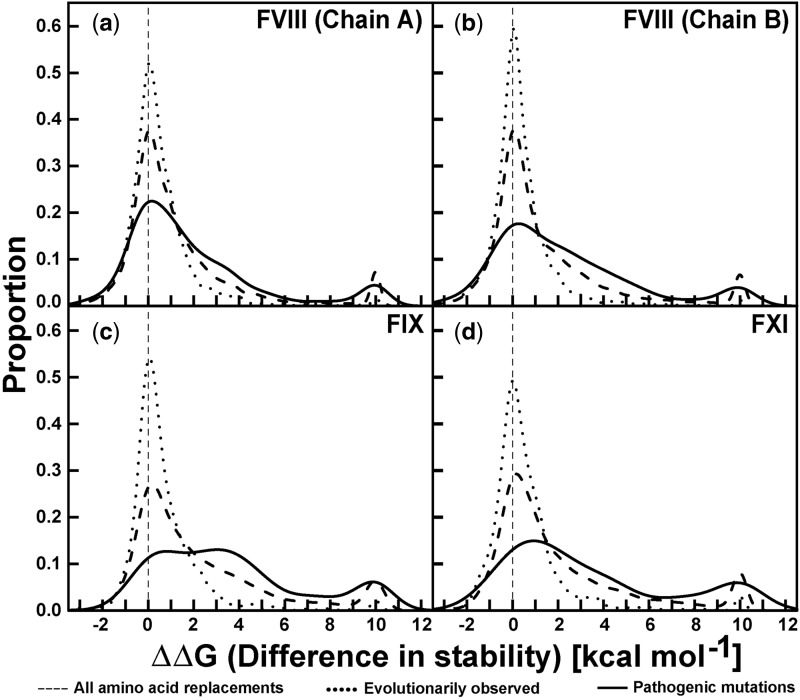
The effect of amino acid replacements on the overall protein stability change ΔΔ*G*. Data are shown for (*a* and *b*) FVIII A and B chains, (*c*) FIX (*d*) FXI. Three calculations were performed, each being based on the sequences for which crystal structures are known (PDB codes: 2R7E [FVIII], 2WPH [FIX] and 2F83 [FXI]). The FVIII A and B chains correspond to the N-terminal residues 19–760 and the C-terminal residues 1582–2351 that are observed in its crystal structure. The calculation represented by the dashed line indicates the distribution calculated for all 19 possible amino acid replacements. That represented by the dotted line indicates the distribution calculated for all the evolutionary occurring replacements that were observed in our data set of 47 genomes. That represented by the continuous line indicates the distribution calculated for the disease-causing mutations from the FVIII, FIX, and FXI mutation databases.

In order to explain the consequences of disease-causing mutations on the protein structure, FoldX software was used to estimate the stability effect (ΔΔ*G*) of residue changes on the three-dimensional structures of human FVIII (chains A and B), FIX, and FXI (fig. 6). FoldX was designed to predict the stability effect of a mutation. This is an empirical method that relies on the estimation of physical parameters from more than 1,000 mutations in protein structures, and has been evaluated with 21 proteins (Schymkowitz et al. 2005; Tokuriki et al. 2007). First, each amino acid at each position was replaced with each of the other 19 amino acids in order to provide a baseline for comparison. As expected, the prediction of all virtual mutations shows a distribution skewed toward destabilization (medians ΔΔ*G* range between 0.51 and 1.23 kcal/mol). Next, the effect of changing the human amino acids with those seen in evolution for the other 46 sequences of this study was calculated in order to examine the effect of evolutionary change on protein stabilities. This produced a narrow distribution predominantly centered on a difference of stability ΔΔ*G* of 0 kcal/mol, and showing a slight skew toward deleterious mutations (medians ΔΔ*G* range between 0.14 and 0.30 kcal/mol). This outcome is as expected because evolutionary changes in the amino acids are selected in order to preserve the stability of the protein structures. Finally, the effect of changing the human amino acids to those observed in unique pathogenic missense mutations was calculated. This analysis produced a clearly skewed distribution (medians ΔΔ*G* range between 1.01 and 2.95 kcal/mol) of ΔΔ*G* values that ranged as high as 10 kcal/mol (and even higher, but these higher values were clipped for easier visualization); this agrees with the fact that these mutations affect the protein structure. The first distribution for the virtual mutations is intermediate between the latter two distributions as expected.

**Fig. 7.**
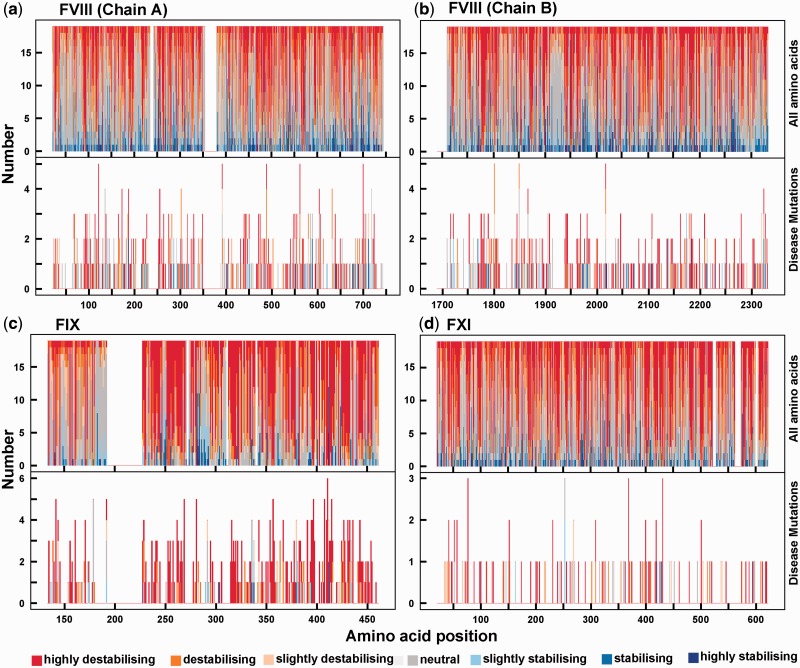
The effect of amino acid replacements at the sequence level on the protein stability change ΔΔ*G*. The residue stability changes are depicted on a seven point scale from highly destabilizing (red) to highly stabilizing (blue) for each of (*a*) FVIII chain A, (*b*) FVIII chain B, (*c*) FIX, and (*d*) FXI. Their PDB codes are indicated in figure 6. In each panel, the upper half shows the ΔΔ*G* values for all the possible 19 amino acid replacements at each residue position that is part of the crystal structure, and the lower half shows the ΔΔ*G* values for the disease-causing mutations taken from the mutation databases.

The same outcome of stability effects was also observed at the individual amino acid level (fig. 7). When the effect of 19 amino acid replacements were computed at each position for FVIII, FIX, and FXI, and the majority of replacements showed a destabilizing effect (>1.0 kcal/mol, in red). A small proportion of replacements stabilized the structure (<−1.0 kcal/mol, in blue), indicating the ability of the protein to compensate deleterious mutations by further mutation. Although this appears to occur more strongly for the FVIII chains A and B (fig. 7*a* and *b*), the proportion of allowed replacements is in fact similar for all four proteins when their different sizes are considered. When the effect of replacements by disease-causing missense mutations was calculated, and the ΔΔ*G* values showed that these almost invariably destabilized the three protein structures.

## Discussion

In this study, we have assembled sequence data for 14 coagulation genes across 47 genomes in order to analyze the evolutionary changes in amino acids that take place between five major clades and several minor ones. These changes were compared directly with disease-causing mutation data for three coagulation proteins FVIII, FIX, and FXI. Good-quality pathogenic mutational data are publicly available for these three proteins, and the bleeding disorders caused by these three sets of mutational data correspond to the three most prevalent bleeding disorders. The outcome of these detailed analyses confirms that positive selection occurred during different stages in the evolution of several coagulation proteins. It was also observed, when comparing the protein structure stabilities, that the pathogenic mutations have a destabilizing effect on the protein structure as opposed to the stable mutations that are tolerated during evolution (fig. 6). The combination of positive selection analyses with those for pathogenic mutations provides information about predicting disease causing mutations and clarifies fresh approaches for therapeutic interventions in bleeding disorders.

### Selective Pressures in Vertebrate Evolution

Evolutionary changes combine random mutational events with natural selection. The probability of a mutation to be fixed in a population depends on its consequence on the fitness of an organism. New adaptive mutations that are beneficial in terms of fitness are more likely to be retained by positive selection than neutral mutation. Neutral mutations that are neither beneficial nor detrimental are randomly retained or removed by genetic drift, depending on the population size (neutral selection). Detrimental mutations are more likely to be removed from the population by negative (or purifying) selection. There are no strict patterns, and a deleterious mutation can sometimes be beneficial to one aspect of the organism. The textbook example of this is sickle-cell anaemia (drepanocytosis) which is a bleeding disorder caused by the p.Glu6Val mutation in the hemoglobin *β* gene. Mutations responsible for sickle-cell anaemia are generally removed by natural selection. In malaria-infested populations, particularly in tropical or subtropical regions, these mutations are selected for in one allele because they offer partial protection against the pathogen (Lopez et al. 2010). The malaria-infected sickle cells inhibit infection by the malaria plasmodium in heterozygous cases (beneficial), but cause sickle-cell anaemia in homozygous cases (deleterious).

Genes corresponding to proteins in direct contact with the environment are more likely to be under adaptive selection. Thus genes involved in host-pathogen interactions are commonly associated with positive selection. Large-scale analyses revealing positive selection in vertebrates or arthropods have been reported for genes involved with immunity (Nielsen et al. 2005; Studer et al. 2008; Montoya-Burgos 2011; Roux et al. 2014). In the present detailed study, positive selection in several genes of the coagulation cascade was identified at different stages in evolution (fig. 3). An earlier study identified positive selection across six mammalian genomes (human, chimpanzee, macaque, mouse, rat, and dog) using LRTs based on codon substitution models and false discovery rates to evaluate multiple LRT comparisons (Kosiol et al. 2008). Although they found strong positive selection (*P* < 0.05 and false discovery rate < 0.05) in nine complement genes, only nominal positive selection (*P* < 0.05) was identified in four coagulation genes (*F2*, *F12*, *TFPI*, *PROC*), none of which were identified in figure 3*b*. A limitation of this earlier analysis may be the low number of six genomes used. Note that complement is related to coagulation in that the latter has the ability to activate complement of innate immunity that mediates host-pathogen interactions (Amara et al. 2008).

### Positive Selection of the Coagulation Proteins through Host-Pathogen Interactions

As a consequence of the immune response, blood coagulation is often exploited by pathogens for reason of infective and septic processes. For example, FG and FV are targeted by bacteria, thus offering a straightforward explanation of positive selection (fig. 3). We discuss this in terms of FG and FII, followed by FIII and FVII, and then FV and FX.

By taking advantage of the increased number of available genome sequences, positive selection has been identified in several coagulation genes during various stages in evolution. In particular, all three subunits FGA, FGB, and FGG of FG have shown positive selection at periods during vertebrate evolution, according to both the site model and the branch-site models. Similarly, prothrombin (FII) has shown positive selection but only across all the vertebrate genomes, and not across any smaller clades. Although the immune system is under strong adaptive constraints because it needs to evolve constantly against pathogens, the coagulation system also comprises the first line of immune defense against bacteria to form a clot to block bacterial invasion (Sun 2006). Both FG and prothrombin (FII) are targeted by certain bacteria. For example, FG is targeted during the course of infection by cysteine protease, a virulence factor of *Streptococcus pyogenes* (Matsuka et al. 1999) and FG binding protein, Efb, of *Staphylococcus aureus*. Similarly, FG-like proteins exist in *Anopheles gambiae* and are involved in the function of the immune system against malaria or bacterial parasites (Dong and Dimopoulos 2009; Lombardo et al. 2013). Thrombin interacts with staphylocoagulase, a protein secreted by the human pathogen *S. aureus* and activates prothrombin without proteolysis to form thrombin. The resulting prothrombin—staphylocoagulase complex binds to FG to cleave this into self-polymerizing fibrin. This process is central to the molecular pathology of *S. aureus* endocarditis because staphylocoagulase bypasses physiological blood coagulation through the evasion of the recognition of prothrombin by circulating thrombin inhibitors (Panizzi et al. 2005).

Positive selection for FIII and FVII is attributed to a host-pathogen interaction with the herpes virus family. These cell surface molecules do not necessarily participate directly in an immune response but can still serve as a convenient gateway for pathogen entry (Vallender and Lahn 2004). Herpes viruses promote cellular surface infections by coevolving with their human host, indicating a mode by which pathogens exploit the coagulation system (Sutherland et al. 2012). A study of the host-pathogen driven coevolution of herpes virus revealed that this virus has existed from the time of mammals and divided into the HSV-1 and HSV-2 types recently during human evolution (Kolb et al. 2013). These observations suggest that the pressure for FIII to evolve in early mammals, especially through primates, could be the reason for the positive selection of FIII across the mammalian clade (fig. 3). We propose that the FIII–FVII interaction is a significant driving force for positive selection in FVII.

The FV and FVIII cofactors are homologous to each other in their sequence and domain organization, hence it is not surprising to see similar selection pressures acting on them (fig. 3*c*). A difference arises in the entire vertebrate genome (fig. 3*a*) where FV shows positive selection whereas FVIII does not. This difference between FV and FVIII across vertebrates may result from the interaction of FV with *Escherichia coli*. The extracellular serine protease EspP from *E. coli* cleaves FV to lead to its degradation, and thus reduces the activity of the coagulation cascade (Brunder et al. 1997). The activated FVa cofactor forms a FVa/FXa complex with activated FX (fig. 1), which is important for thrombin formation, the most important step for fibrin clot formation. Because both FVa and FXa are central to coagulation, and are associated with bacterial proteolysis, this offers an explanation for the signatures of positive selection in both proteins.

FVIII, FIX, and FXIII are also associated with positive selection; however, it is not clear what selective pressures are involved. Although these proteins may also be targeted by pathogens, no direct interactions have been reported so far.

#### Absence of Positive Selection for FXI and FXII

FXII is unique in that it does not show positive selection, even though it is involved in host-pathogen interactions with long-chain inorganic phosphatases (polyP) from microorganisms (Morrissey et al. 2012). Small-chain polyP are produced in the human brain and by blood platelets. Hence, even during the absence of pathogen-originated polyP, the human host does not lack polyP and there is no pressure to evolve for this (Puy et al. 2013). A *F12* deficiency in patients does not cause bleeding disorders. This means that the coagulation cascade can be initiated even in the absence of *F12*. For similar reasons, FXI shows no positive selection. We hypothesize that this is because, first, no interaction with the environment has been demonstrated to date, and, second, with respect to the polyP mechanism, the coagulation cascade is capable of activating in a FXI-independent manner.

### Relationship between Disease-Causing Mutations and Selection

The mechanisms of genetic drift, mutational change, and evolutionary change are closely related to each other. Evolutionary change is not only dependent on the genetic variability from mutational change, but also on genetic drift, which describes the random fluctuations in the number of genetic variants in an organism. Genetic drift and mutations produce random variations that selection can act upon. The new changes incurred in one generation may be cancelled in another generation. It may, therefore, be important to determine whether a new mutation is lost or becomes common enough for selection to determine its fate (Masel 2011). In this study, we have examined the relationship between natural selection acting on an amino acid and the disease-causing mutations reported at that amino acid position for the three prominent coagulation factors FVIII, FIX, and FXI. We have shown that the disease-causing mutations are more likely to occur at a higher frequency at sites under negative selection in all three proteins (fig. 5). As sites under strong selective pressure are more likely to be critical to protein function, such a mutation can readily affect protein function and trigger disease. Lethal (or deleterious) mutations that cause death are de facto not observed in our data set. By contrast, disease-causing mutations occur at a reduced frequency in sites under neutral or positive selection, suggesting that these sites are more tolerant to mutations than sites under negative selection. Interestingly, we have shown that FVIII and FIX have undergone much positive selection whereas FXI has no such signatures (fig. 4). Despite this difference, our results for three proteins show no change in the relationship between evolutionary selection and disease-causing mutations (figs. 5 and 6). In summary, the reported disease-causing mutations show randomness, and the mutability of a given amino acid position depends on the selection pressure acting at that position and not on the entire protein.

### Effect of Disease-Causing Mutations on the Stability of Protein Structure

Mutations that change the amino acid sequence can have striking effects on protein stability. Point mutations are related to pathological and genetic conditions by reducing or abolishing coagulation protein function (table 1). Protein function is related to its stability which is often measured by the change in folding energy ΔΔ*G* of the protein structure (Stefl et al. 2013). We have identified the effect of mutations on the stability of FVIII, FIX, and FXI in terms of ΔΔ*G* values. The stability effects of polymorphic amino acid changes in human sequences follow a Gaussian distribution (de Beer et al. 2013). In that study, disease-causing mutations also showed the same distribution, but slightly skewed toward destabilization. In this study, the same trend for amino acids substitutions in vertebrates were observed, but this is much stronger for disease-causing mutations (fig. 6). The difference between the two studies may be explained by the different methods used. de Beer et al. (2013) used the Discrete Optimized Protein (DOPE) score of Modeller as a monitor of global stability, whereas we used FoldX (Materials and Methods) as an empirical method to infer directly the stability effect ΔΔ*G* of a point mutation.

Evolutionarily observed mutations are the most stable in terms of their protein structure, whereas disease-causing mutations destabilized the protein structure (figs. 6 and 7). Each site may or may not tolerate a particular amino acid change, depending on the change in stability. When each amino acid was converted to each of the other 19 possible amino acids (fig. 7, top panels), the ΔΔ*G* values showed that most amino acids destabilize the structure whereas several may stabilize it. The disease-causing effect of a mutation may be affected, not only by the selection pressure acting on it, but also by the type of the amino acid replaced. For the FVIII, FIX, and FXI bleeding disorders, destabilization was observed for most of the disease-causing mutations.

### Utility of Evolutionary and Disease-Causing Mutational Analyses for Clinical Diagnosis

Interactive locus-specific databases are useful tools for presenting patient mutational data for patient diagnosis and care. We plan to incorporate the above data sets on selective pressures and stability effects into our mutational databases for FVIII, FIX, and FXI. This information will provide insight for the prediction of the effect of disease-causing mutations. The level of selective pressure was estimated under a maximum-likelihood framework. Such methods are useful for genetic studies but their results are sometimes difficult to interpret by a nonspecialist. The presentation of this information needs to be intuitive to the viewer. As shown in supplementary figure S3, Supplementary Material online, the use of multiple sequence alignments and presentation of stability effects ΔΔ*G*, structural locations and selective pressures can help in interpreting the impacts of the mutations. This type of information will therefore clarify the interpretation of newly discovered disease-causing mutations for clinicians, evolutionary biologists, and protein biochemists. This combined evolutionary-driven study of bleeding disorder-causing mutations is the most detailed of its kind as far as we are aware, and may be usefully extended to other biological processes and their proteins.

## Materials and Methods

### Genomic Sequences

The flowchart of operations is summarized in supplementary figure S1, Supplementary Material online. First, the complete multiple sequence alignments were retrieved, together with their gene trees, for each of the 14 genes corresponding to the 11 coagulation factors (table 1), from the Ensembl Compara database (Vilella et al. 2009) using their Ensembl IDs (table 1) and a Perl script. Ensembl release 73 was used, accessed on September 30, 2013 (Flicek et al. 2013). These amino acid sequences came from Ensembl, as they are generally of good quality, and contained 74 vertebrate genomes in a total of 80 genomes. We then excluded any sequences that came from genomes of low coverage (2× or less). This resulted in 47 vertebrate genomes with alignment sizes between 23 and 47 genomes. These 47 sets of vertebrate sequences provided sufficient genomic coverage to perform phylogenetic analysis. The 47 vertebrate organisms were classified into five major clades (fig. 2), namely Primates, Glires (rodents, rabbits, and pika), Laurasiatheria*,* Sauropsida (reptiles and birds), and Fishes (ray-finned fishes), together with minor clades including Atlantogenata and Australian mammals.

### Sequence Retrieval and Multiple Alignments

The multiple sequence alignments and gene trees from EnsemblCompara included all the coagulation sequences in different species, irrespective of their genome coverage. The multiple sequence alignments of the downloaded genes from the EnsemblCompara database were constructed using a global alignment algorithm provided within this database for significant number of sequences of arbitrary lengths. The data set required for this study comprised 14 genes from 47 genomes. A thorough quality control procedure of the multiple sequence alignments was performed in order to generate minimum false positive results. Thus sequences were removed if they showed >1% low-complexity regions or unknown nucleotides (indicated as “N” in a sequence). From these, the phylogenetic trees were pruned using Newick Utilities software (Junier and Zdobnov 2010) to identify the tips corresponding to the retrieved species (fig. 2). The final alignments of these sequences were made with PRANK, which is conservative in that it tends to align only amino acids derived by substitution and introduces insertions and deletions (indels) instead of aligning sequence fragments that appear too divergent (Loytynoja and Goldman 2008). PRANK is an advanced probabilistic alignment algorithm that incorporates phylogenetic information. This has been shown to reduce the number of false-positives when evaluating positive selection (Fletcher and Yang 2010; Loytynoja 2014). All the PRANK alignments were made using default parameters for the empirical codon model and the pruned trees as guides. The resulting alignments are composed of highly conserved blocks surrounded by noise due to indels. Finally Gblocks (Castresana 2000) was used to remove the noise in the unreliable regions of the multiple sequence alignments which may cause false positives when testing for positive selection, to keep only the well-aligned parts. The default Gblocks parameters for the codontype model (parameter –t = c) were used, with the exception of tolerating half gaps per position (parameter -b5=h). This procedure resulted in clean and accurate multiple sequence alignments for the final computations of the site and branch-site models using CodeML together with their corresponding gene trees.

### Analyses of Evolutionary Pressure

For each of the 14 coagulation genes within each of the five major clades, the selective pressures were estimated using the different codon substitution site models implemented in CodeML of the phylogenetic analysis with maximum-likelihood software (PAML release 4.7) (Yang 2007). By analyzing the protein-coding genes at the nucleotide level, the synonymous or silent changes (nucleotide substitutions that do not change the encoded amino acid) are distinguished from the nonsynonymous or missense changes (substitutions that change the encoded amino acid). Because natural selection operates at the protein level, and can take any direction of positive, neutral or negative selection, the synonymous and nonsynonymous substitutions are subjected to different selective pressures which occur at the rates d_S_ and d_N_, respectively. Hence, the ratio of nonsynonymous/synonymous substitution rates (*ω* = *d*_N_/*d*_S_) measures the selective pressure at the protein level. The value of *ω* reveals the direction and strength of natural selection acting on the protein. Values of *ω* < 1, *ω* = 1 and *ω* > 1 indicate negative selection, neutral evolution, and positive selection, respectively. If an existing function or phenotype is evolutionarily favorable, then negative selection (or purifying selection) favors the conservation of that function or phenotype (conservation of amino acids). The other is positive selection (or Darwinian selection) which favors the promotion of a new function or phenotype. Neutral evolution describes the tolerance to amino acid mutations, which are neither deleterious nor beneficial, and are fixed according to genetic drift.

The following five models were used to account for the variation of *d*_N_/*d*_S_ among codon sites: M1a (two *d*_N_/*d*_S_ ratios with negative selection [*ω*_0_ < 1] and neutral evolution [*ω*_1_ = 1]); M2a (three *d*_N_/*d*_S_ ratios with negative selection [*ω*_0_ < 1], neutral evolution [*ω*_1_ = 1] and positive selection [*ω*_2_ ≥ 1]), M7 (beta); M8 (beta and *ω*_2_ > 1); and M8a (beta and *ω*_2_ = 1). The alignments contain several positions in which one of the sequences is missing, and the “cleandata = 0” option was selected to retain all sites. The inference of positive selection was conducted by performing LRTs in which the following two pairs of models were compared, namely M1a with M2a, and M8a with M8. The principle is to compare a null model that does not allow *ω*_2_ > 1 (M1a or M8a) with an alternative model that does (M2a or M8). Because the alternate model has more parameters than the null model, the LRT follows a χ^2^ distribution. The LRT is a powerful method for comparing two hypotheses where one is the special case of the other. The BEB method (Yang et al. 2005) was used to calculate the posterior probabilities for site classes, and the BEB value was used to identify sites under positive selection where the χ^2^ distribution of the LRT was significant with *P* < 0.05. As many different LRT comparisons (different genes × different clades) were conducted, a control for the false-discovery rate (expected proportion of false positives) was applied using the *q* value package in R (Storey and Tibshirani 2003). We adjusted the parameters of the *q* value to perform a bootstrap analysis (pi0.meth = “bootstrap”) and to tolerate 10% of false positives in our significant results (“fdr.level = 0.1”). A bootstrap analysis is necessary as the *P* value distribution is bimodal (Studer et al. 2008).

Two sources for the branch-site model were used. First, the Selectome database at http://selectome.unil.ch/ (last accessed January 2014) (Proux et al. 2009; Moretti et al. 2014) provides the precomputed results of the branch-site model (supplementary table S2*b*, Supplementary Material online), based on the multiple sequence alignments from the Ensembl Compara database (Vilella et al. 2009). The last release of Selectome (release 6) contains an optimized procedure of alignment refinement and quality control, similar to the one used for our site-model analyses, as described above. The branch-site model allows the *d*_N_/*d*_S_ value to vary, not only between sites, but also between branches (Zhang et al. 2005). The advantage of this model is its ability to detect traces of episodic positive selection (Studer and Robinson-Rechavi 2009a). Second, a branch-site model was determined based on our data set (see above). We used the vertebrate trees and their multiple sequence alignments as inputs for branch-site model calculations. The multiple alignments were manually rechecked to remove barren sequences, and the corresponding vertebrate trees were edited accordingly using Newick Utilities. Each taxonomic unit, also known as the branch or node of the tree, was tagged using an automated python script. The CodeML program was run on each of these tagged trees from all the genes for this branch-site model (supplementary table S2*b*, Supplementary Material online). For some alignments, we found misaligned short insertions up to a maximum of ten amino acids in the conserved blocks, probably due to gene model prediction errors. In our whole data set, these errors affect 0–4 sequences per alignment. As the *F2* gene family contained most of these small errors, albeit at a very low level, this was reanalyzed by removing four sequences with potential problems from the alignment. The recalculation of the likelihood ratio deviated only slightly from the initial results (*P* value of 3.93e^−^^5^ for 38 sequences vs. *P* value of 2.17e^−^^4^ for 34 sequences). This suggested that these errors would only have a minor impact, if any, on the detection of positive selection, especially as the coagulation factor sequences were long, and the model of codon substitutions was averaged over all columns (many hundreds) and sequences (between 23 and 47).

### Stability Effect of Mutations

When possible for each coagulation factor, the molecular structures of the domain(s) were retrieved directly from the CATH database (Sillitoe et al. 2013). Prior to the mutational analyses, each structure was repaired with the FoldX program (Schymkowitz et al. 2005) to remove potential steric clashes. FoldX utilizes an empirical force field model to estimate the stability of a protein _(_ΔG_wildtype_ in kcal/mol). FoldX was used to estimate the stability effect on each residue position when the residue was replaced by any of the 19 other amino acids. The stability effect is determined by the difference in the wildtype and mutant energies (ΔΔ*G* = Δ*G*_mutant_ − Δ*G*_wildtype_). This resulted in a stability landscape of all potential mutations, including the ΔΔ*G* values both for the disease mutations and for all the amino acid substitutions observed across vertebrate evolution.

### Statistical Analyses

All statistical analyses were performed with R v3.0.2 (R Core Team 2013) and SigmaPlot (Systat Software, San Jose, CA). The pipeline was developed with Biopython scripts (Cock et al. 2009). The visualization of the multiple sequence alignments was performed using Jalview 2.8 (Waterhouse et al. 2009).

### Data Availability

The final sequence alignments are provided on a webpage http://www.biochem.ucl.ac.uk/pavithra/evolution/coagulation/index.php (last accessed January 2014). Pathogenic mutation data for three human coagulation factors FVIII, FIX, and FXI were retrieved from our web databases at http://www.factorviii-db.org/, http://www.factorix.org/, and http://www.factorxi.org/ (last accessed January 2014) (Saunders et al. 2009; Rallapalli et al. 2013).

## Supplementary Material

Supplementary tables S1–S2 and figures S1–S3 are available at *Molecular Biology and Evolution* online (http://www.mbe.oxfordjournals.org/).

Supplementary Data
