# Supplementary material for: Positive Selection during the Evolution of the Blood Coagulation Factors in the Context of Their Disease-Causing Mutations
Source: Mol Biol Evol. 2014 Aug 25;31(11):3040–56. doi: 10.1093/molbev/msu248 (PMC4209140; doi:10.1093/molbev/msu248)
Supplement: Supplementary Data [file supp_31_11_3040__index.html]

Positive selection during the evolution of the blood coagulation factors in the context of their disease-causing mutations — Positive Selection during the Evolution of the Blood Coagulation Factors in the Context of Their Disease-Causing Mutations — Positive Selection during the Evolution of the Blood Coagulation Factors in the Context of Their Disease-Causing Mutations — Supplementary Data 

# Positive Selection during the Evolution of the Blood Coagulation Factors in the Context of Their Disease-Causing Mutations

## Supplementary Data

files

**Files in this Data Supplement:**

- Supplementary Data - pdf file
- Supplementary Data - pdf file
- Supplementary Data - pdf file
