# Supplementary figures and images for: Positive Selection during the Evolution of the Blood Coagulation Factors in the Context of Their Disease-Causing Mutations
Source: Mol Biol Evol. 2014 Aug 25;31(11):3040–56. doi: 10.1093/molbev/msu248 (PMC4209140; doi:10.1093/molbev/msu248)

## (a) FVIII - Part I

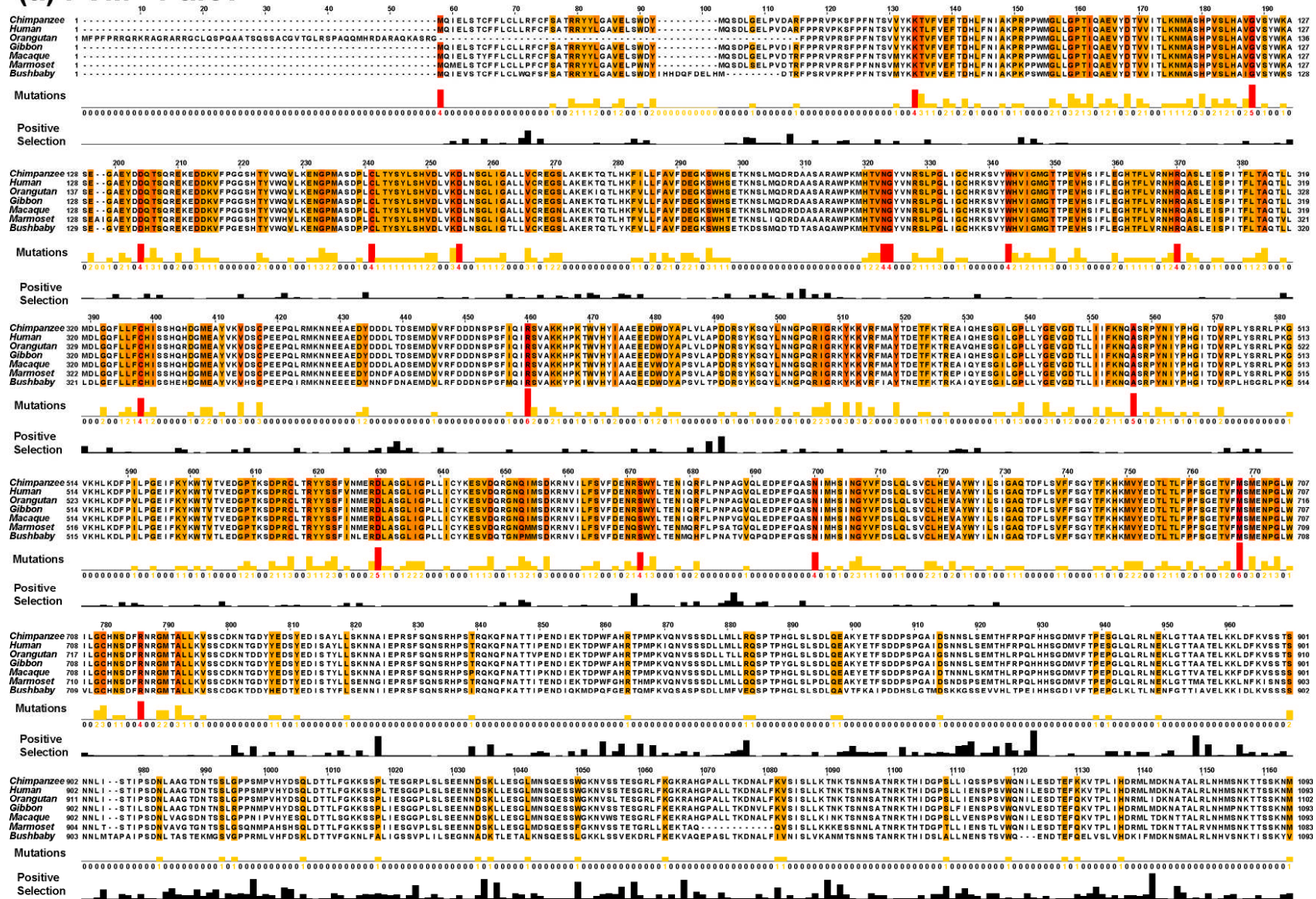

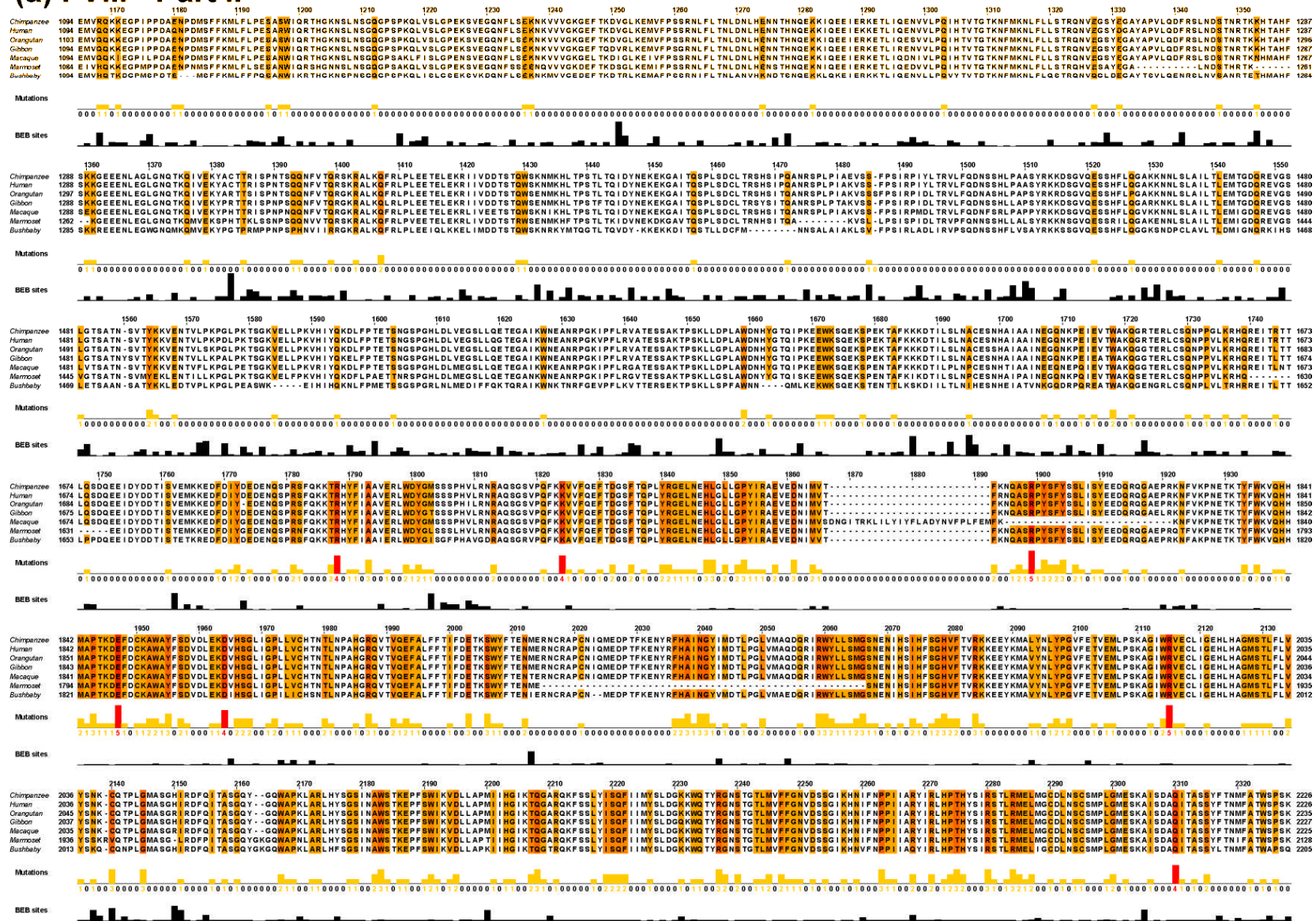

(a) FVIII - Part III

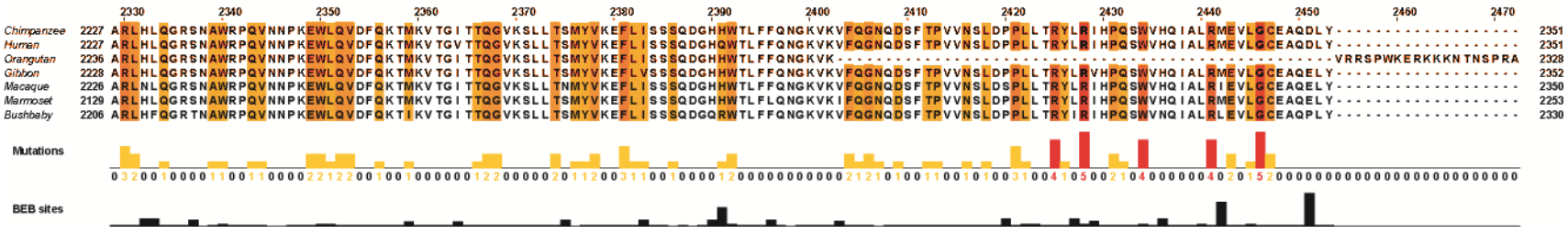

FIGURE S3(a)

Supplement: Supplementary Data [file supp_msu248_Positive-Selection_supplementary_material_S3a.pdf]

## (b) FIX

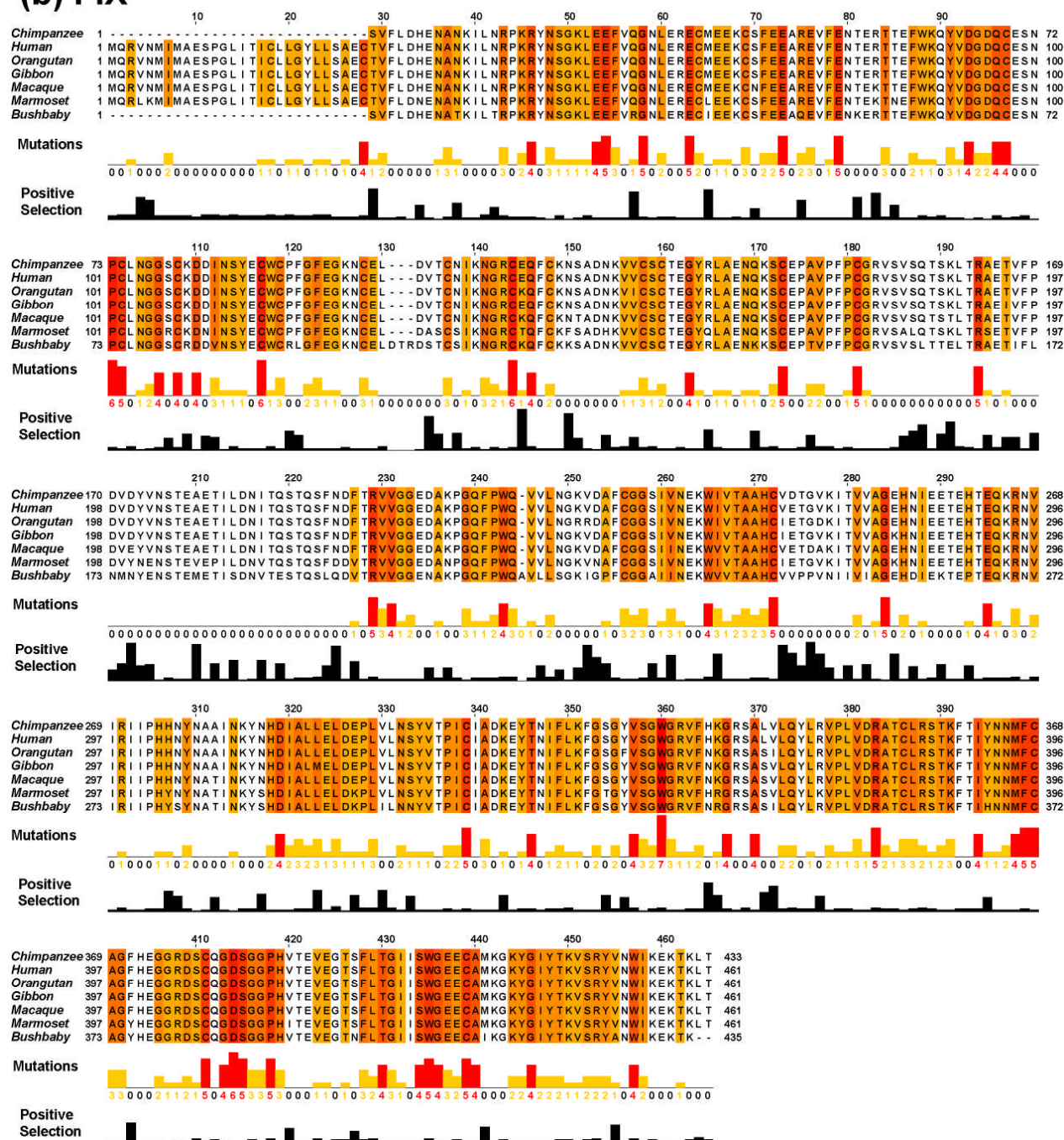

FIGURE S3(b)

### (c) FXI

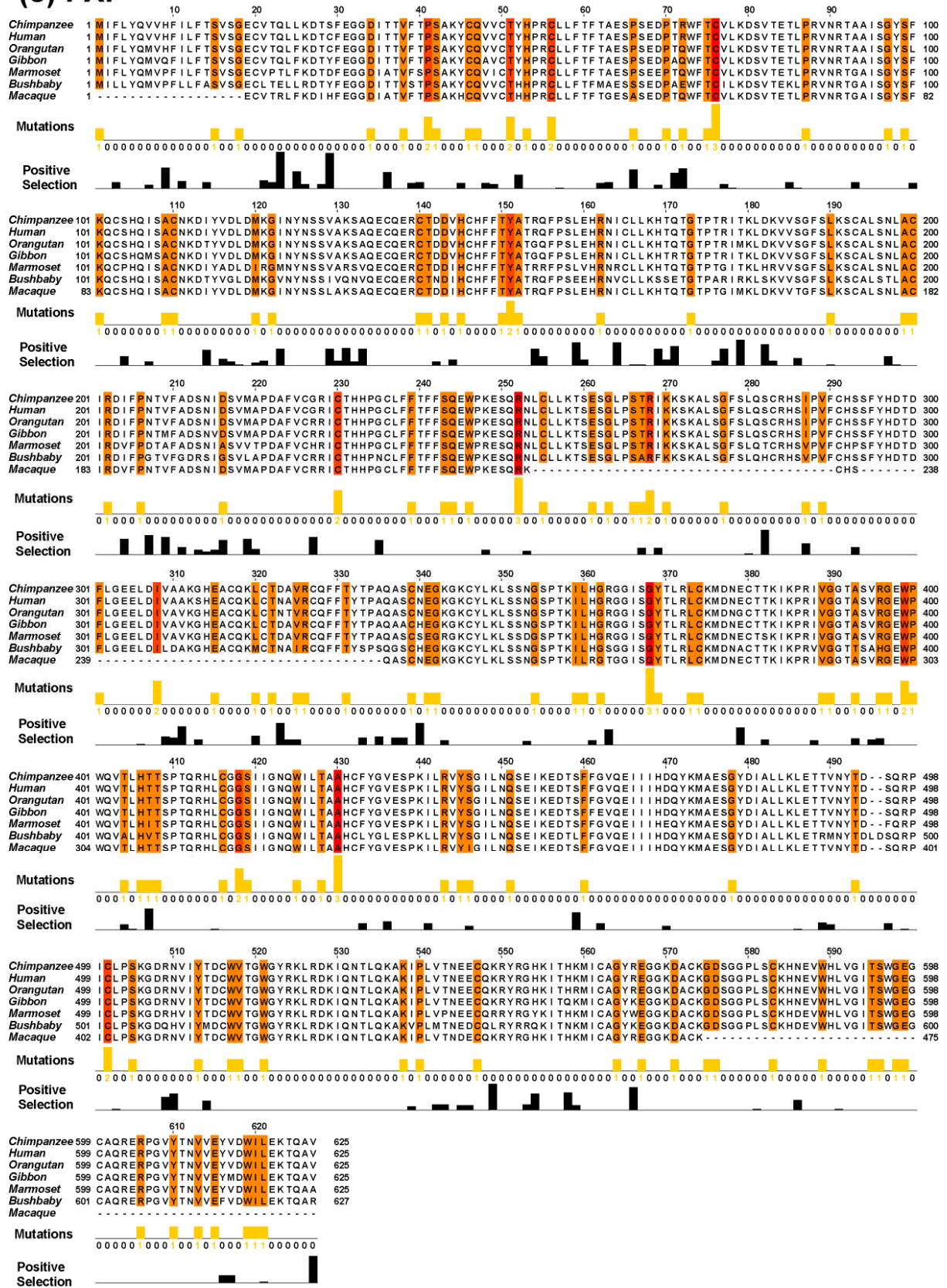

FIGURE S3(c)

Supplement: Supplementary Data [file supp_msu248_Positive-Selection_supplementary_material_S3b_S3c.pdf]
