# Supplementary material for: Positive Selection during the Evolution of the Blood Coagulation Factors in the Context of Their Disease-Causing Mutations
Source: Mol Biol Evol. 2014 Aug 25;31(11):3040–56. doi: 10.1093/molbev/msu248 (PMC4209140; doi:10.1093/molbev/msu248)
Supplement: Supplementary Data [file supp_msu248_Positive-Selection_supplementary_material_Tables_S1_S2.pdf]

SUPPLEMENTARY MATERIAL

Table S1: Presence and absence of the 14 coagulation genes in each of the 47 genomes used in this study.

Clades	Scientific name	Common name	FGA	FGB	FGG	F2	F3	F5	F7	F8	F9	F10	F11	F12	F13A	F13B
Primates	*Callithrix jacchus*	Marmoset	Yes	Yes	Yes	Yes	Yes	Yes	Yes	Yes	Yes	Yes	Yes	Yes	Yes	Yes
Primates	*Homo sapiens*	Human	Yes	Yes	Yes	Yes	Yes	Yes	Yes	Yes	Yes	Yes	Yes	Yes	Yes	Yes
Primates	*Macaca mulatta*	Macaque	Yes	Yes	Yes	Yes	Yes	Yes	Yes	Yes	Yes	Yes	Yes	Yes	Yes	Yes
Primates	*Nomascus leucogenys*	Gibbon	Yes	Yes	Yes	Yes	Yes	Yes	Yes	Yes	Yes	Yes	Yes	Yes	Yes	Yes
Primates	*Otolemur garnettii*	Bushbaby	Yes	Yes	Yes	Yes	Yes	Yes	Yes	Yes	Yes	Yes	Yes	Yes	Yes	Yes
Primates	*Pongo abelii*	Orangutan	Yes	Yes	Yes	Yes	Yes	Yes	Yes	Yes	Yes	Yes	Yes	Yes	Yes	Yes
Primates	*Pan troglodytes*	Chimpanzee	Yes	Yes	Yes	Yes	Yes	Yes	Yes	Yes	Yes	Yes	Yes	Yes	Yes	Yes
Glires	*Cavia porcellus*	Guinea Pig	Yes	Yes	Yes	Yes	Yes	Yes	Yes	Yes	Yes	Yes	Yes	Yes	Yes	Yes
Glires	*Dipodomys ordii*	Kangaroo rat	Yes	Yes	Yes	Yes	Yes	Yes	Yes	Yes	Yes	Yes	Yes	Yes	Yes	Yes
Glires	*Mus musculus*	Mouse	Yes	Yes	Yes	Yes	Yes	Yes	Yes	Yes	Yes	Yes	Yes	Yes	Yes	Yes
Glires	*Oryctolagus cuniculus*	Rabbit	Yes	Yes	Yes	Yes	Yes	Yes	Yes	Yes	Yes	Yes	Yes	Yes	Yes	Yes
Glires	*Ochotona princeps*	Pika	Yes	Yes	Yes	Yes	Yes	Yes	Yes	Yes	Yes	Yes	Yes	Yes	Yes	Yes
Glires	*Rattus norvegicus*	Rat	Yes	Yes	Yes	Yes	Yes	Yes	Yes	Yes	Yes	Yes	Yes	Yes	Yes	Yes
Glires	*Ictidomys tridecemlineatus*	Squirrel	Yes	Yes	Yes	Yes	Yes	Yes	Yes	Yes	Yes	Yes	Yes	Yes	Yes	Yes
Laurasiatheria	*Ailuropoda melanoleuca*	Panda	Yes	Yes	Yes	Yes	Yes	Yes	Yes	Yes	Yes	Yes	Yes	Yes	Yes	Yes
Laurasiatheria	*Bos taurus*	Cow	Yes	Yes	Yes	Yes	Yes	Yes	Yes	Yes	Yes	Yes	Yes	Yes	Yes	Yes
Laurasiatheria	*Canis lupus familiaris*	Dog	Yes	Yes	Yes	Yes	Yes	Yes	Yes	Yes	Yes	Yes	Yes	Yes	Yes	Yes
Laurasiatheria	*Equus caballus*	Horse	Yes	Yes	Yes	Yes	Yes	Yes	Yes	Yes	Yes	Yes	Yes	Yes	Yes	Yes
Laurasiatheria	*Felis catus*	Cat	Yes	Yes	Yes	Yes	Yes	Yes	Yes	Yes	Yes	Yes	Yes	Yes	Yes	Yes
Laurasiatheria	*Mustela putorius furo*	Ferret	Yes	Yes	Yes	Yes	Yes	Yes	Yes	Yes	Yes	Yes	Yes	Yes	Yes	Yes
Laurasiatheria	*Sus scrofa*	Pig	Yes	Yes	Yes	Yes	Yes	Yes	Yes	Yes	Yes	Yes	Yes	Yes	Yes	Yes
Atlantogenata	*Choloepus hoffmanni*	Sloth	Yes	Yes	Yes	**No**	Yes	Yes	Yes	**No**	**No**	Yes	Yes	**No**	Yes	Yes
Atlantogenata	*Dasypus novemcinctus*	Armadillo	Yes	Yes	Yes	Yes	Yes	Yes	Yes	Yes	Yes	Yes	Yes	Yes	Yes	Yes

Atlantogenata	*Echinops telfairi*	Lesser hedgehog tenrec	Yes	Yes	Yes	Yes	**No**	Yes	Yes	Yes	Yes	Yes	Yes	Yes	Yes	Yes
Atlantogenata	*Loxodonta africana*	Elephant	Yes	Yes	Yes	Yes	Yes	Yes	Yes	Yes	Yes	Yes	Yes	Yes	Yes	Yes
Atlantogenata	*Procavia capensis*	Hyrax	Yes	Yes	Yes	Yes	Yes	Yes	Yes	Yes	Yes	Yes	Yes	Yes	Yes	Yes
Mammals	*Macropus eugenii*	Wallaby	Yes	Yes	Yes	Yes	Yes	Yes	Yes	Yes	Yes	Yes	Yes	Yes	Yes	Yes
Mammals	*Monodelphis domestica*	Opossum	Yes	Yes	Yes	Yes	Yes	Yes	Yes	Yes	Yes	Yes	Yes	Yes	Yes	Yes
Mammals	*Ornithorhynchus anatinus*	Platypus	Yes	Yes	Yes	Yes	Yes	Yes	Yes	Yes	Yes	Yes	**No**	Yes	Yes	Yes
Mammals	*Sarcophilus harrisii*	Tasmanian devil	Yes	Yes	Yes	Yes	Yes	Yes	Yes	Yes	Yes	Yes	Yes	Yes	Yes	Yes
Sauropsida	*Anolis carolinensis*	Anole lizard	Yes	Yes	Yes	Yes	Yes	Yes	Yes	Yes	Yes	Yes	**No**	**No**	Yes	Yes
Sauropsida	*Anas platyrhynchos*	Duck	Yes	Yes	Yes	Yes	Yes	Yes	Yes	Yes	Yes	Yes	**No**	**No**	Yes	Yes
Sauropsida	*Ficedula albicollis*	Flycatcher	Yes	Yes	Yes	Yes	Yes	Yes	Yes	Yes	Yes	Yes	**No**	**No**	Yes	Yes
Sauropsida	*Gallus gallus*	Chicken	Yes	Yes	Yes	Yes	Yes	Yes	Yes	Yes	Yes	Yes	**No**	**No**	Yes	Yes
Sauropsida	*Meleagris gallopavo*	Turkey	Yes	Yes	Yes	Yes	Yes	Yes	Yes	Yes	Yes	Yes	**No**	**No**	Yes	Yes
Sauropsida	*Pelodiscus sinensis*	Chinese softshell turtle	Yes	Yes	Yes	Yes	Yes	Yes	Yes	Yes	Yes	Yes	**No**	**No**	Yes	Yes
Sauropsida	*Taeniopygia guttata*	Zebra Finch	Yes	Yes	Yes	Yes	Yes	Yes	Yes	Yes	Yes	Yes	**No**	**No**	Yes	Yes
Other	*Latimeria chalumnae*	Coelacanth	Yes	Yes	Yes	Yes	Yes	Yes	Yes	Yes	Yes	Yes	**No**	Yes	Yes	Yes
Tetrapoda	*Xenopus tropicalis*	Xenopus	Yes	Yes	Yes	Yes	Yes	Yes	Yes	Yes	Yes	Yes	**No**	Yes	Yes	Yes
Fishes	*Danio rerio*	Zebrafish	Yes	Yes	Yes	Yes	Yes	Yes	Yes	Yes	Yes	Yes	**No**	**No**	Yes	**No**
Fishes	*Gasterosteus aculeatus*	Stickleback	Yes	Yes	Yes	Yes	Yes	Yes	Yes	Yes	Yes	Yes	**No**	**No**	Yes	**No**
Fishes	*Gadus morhua*	Cod	Yes	Yes	Yes	Yes	Yes	Yes	Yes	Yes	Yes	Yes	**No**	**No**	Yes	**No**
Fishes	*Oreochromis niloticus*	Tilapia	Yes	Yes	Yes	Yes	Yes	Yes	Yes	Yes	Yes	Yes	**No**	**No**	Yes	**No**
Fishes	*Oryzias latipes*	Medaka	Yes	Yes	Yes	Yes	Yes	Yes	Yes	Yes	**No**	Yes	**No**	**No**	Yes	**No**
Fishes	*Tetraodon nigroviridis*	Tetraodon	Yes	Yes	Yes	Yes	Yes	Yes	Yes	Yes	Yes	Yes	**No**	**No**	Yes	**No**
Fishes	*Takifugu rubripes*	Fugu	Yes	Yes	Yes	Yes	Yes	Yes	Yes	Yes	Yes	Yes	**No**	**No**	Yes	**No**
Fishes	*Xiphophorus maculatus*	Platyfish	Yes	Yes	Yes	Yes	Yes	Yes	Yes	Yes	Yes	Yes	**No**	**No**	Yes	**No**

Table S2a: Positively selected genes of the coagulation cascade within different groups of species as calculated with the site model in this study.

	Vertebrates	Fishes	Sauropsida	Mammals	Laurasiatheria	Glires	Primates
FGA	No	No	No	**Yes**	**Yes**	No	No
FGB	No	No	No	No	No	No	**Yes**
FGG	No	No	No	No	No	No	No
F2	**Yes**	No	No	No	No	No	No
F3	No	No	**Yes**	**Yes**	No	No	No
F5	**Yes**	No	No	**Yes**	No	No	**Yes**
F7	**Yes**	No	No	No	No	No	No
F8	No	No	No	**Yes**	No	No	**Yes**
F9	No	No	No	**Yes**	No	No	No
F10	**Yes**	No	No	No	No	No	No
F11	No	No	No	No	No	No	No
F12	No	No	No	No	No	No	No
F13A	**Yes**	No	No	No	No	No	No
F13B	No	No	No	No	**Yes**	No	No

Table S2b: Positively selected genes of the coagulation cascade in different branches of the taxonomic tree of vertebrates as calculated in the branch-site model in this study. Within brackets () are given the results from the Selectome Database for comparison. (-) means that the results for the gene family are not available in Selectome.

Gene	Codons analysed	Actinopterygii	Sarcopterygii	Tetrapoda	Amniota	Sauropsida	Mammals	Theria	Eutheria
FGA	135	**Yes** (No)	**Yes** (No)	No (No)	No (**Yes**)	No (No)	No (No)	No (No)	No (No)
FGB	315	**Yes** (**Yes**)	**Yes** (**Yes**)	**Yes** (No)	**Yes** (**Yes**)	**Yes** (No)	**Yes** (**Yes**)	No (**Yes**)	No (No)
FGG	299	No (No)	No (No)	No (No)	No (No)	No (No)	No (**Yes**)	No (No)	No (No)
F2	391	**Yes** (No)	**Yes** (No)	**Yes** (**Yes**)	**Yes** (**Yes**)	No (No)	**Yes** (No)	No (-)	**Yes** (**Yes**)
F3	122	No (No)	No (No)	No (No)	No (No)	No (No)	No (No)	No (No)	No (No)
F5	488	**Yes** (-)	**Yes** (-)	**Yes** (-)	**Yes** (-)	No (-)	No (-)	No (-)	No (-)
F7	126	(-) (Yes)	(-) (**Yes**)	No (No)	No (No)	No (No)	No (No)	No (No)	No (No)
F8	701	**Yes** (-)	**Yes** (-)	**Yes** (-)	**Yes** (-)	**Yes** (-)	**Yes** (-)	No (-)	No (-)
F9	218	**Yes** (**Yes**)	**Yes** (**Yes**)	No (**Yes**)	**Yes** (**Yes**)	**Yes** (**Yes**)	No (No)	No (No)	**Yes** (**Yes**)
F10	197	No (**Yes**)	No (**Yes**)	No (**Yes**)	**Yes** (**Yes**)	No (**Yes**)	No (**Yes**)	No (No)	No (No)
F11	152	No (-)	No (-)	No (-)	No (-)	No (-)	No (-)	No (-)	No (-)
F12	306	No (-)	No (-)	No (-)	**Yes (-)**	No (-)	**Yes** (-)	No (-)	No (-)
F13A	388	**Yes** (**Yes**)	**Yes** (**Yes**)	**Yes** (**Yes**)	**Yes** (No)	No (No)	No (No)	No (**Yes**)	No (No)
F13B	245	(-) (-)	(-) (-)	No (-)	**Yes** (-)	No (-)	No (-)	No (-)	No (-)

Figure Legends

Supplementary Figure S1. Schematic representation of the workflow of this study. The programs (Methods) and the order in which they were employed to provide the multiple sequence alignments of sufficient quality and the phylogenetic trees is shown. This procedure enabled the calculation of selective pressures using CodeML.

Supplementary Figure S2. The probabilities of negative, neutral and positive selection were plotted against the amino acid position for the other 11 coagulation genes of the 14 across the Primates. This extends Figure 4 in the main text in which only the three genes for FIX, FVIII and FXI are shown. The probability values were obtained from CodeML analyses of the seven primate sequences (grey, negative selection; yellow, neutral selection; green, positive selection).

Supplementary Figure S3. Summary of the seven *Primates* sequences to show the degree of positive selection during evolution and the pathogenic mutations at each site for (a) FVIII, (b) FIX, and (c) FXI. The point mutations in the coding regions were obtained from the disease-causing databases for (a) FVIII, (b) FIX, and (c) FXI. Red and orange denote ≥4 mutations and 1-3 mutations respectively. The probabilities of positive selection were taken from the CodeML runs for the seven primate sequences. Because FVIII is a large protein with 2351 residues, the figure was divided into three parts to accommodate the sequence length.

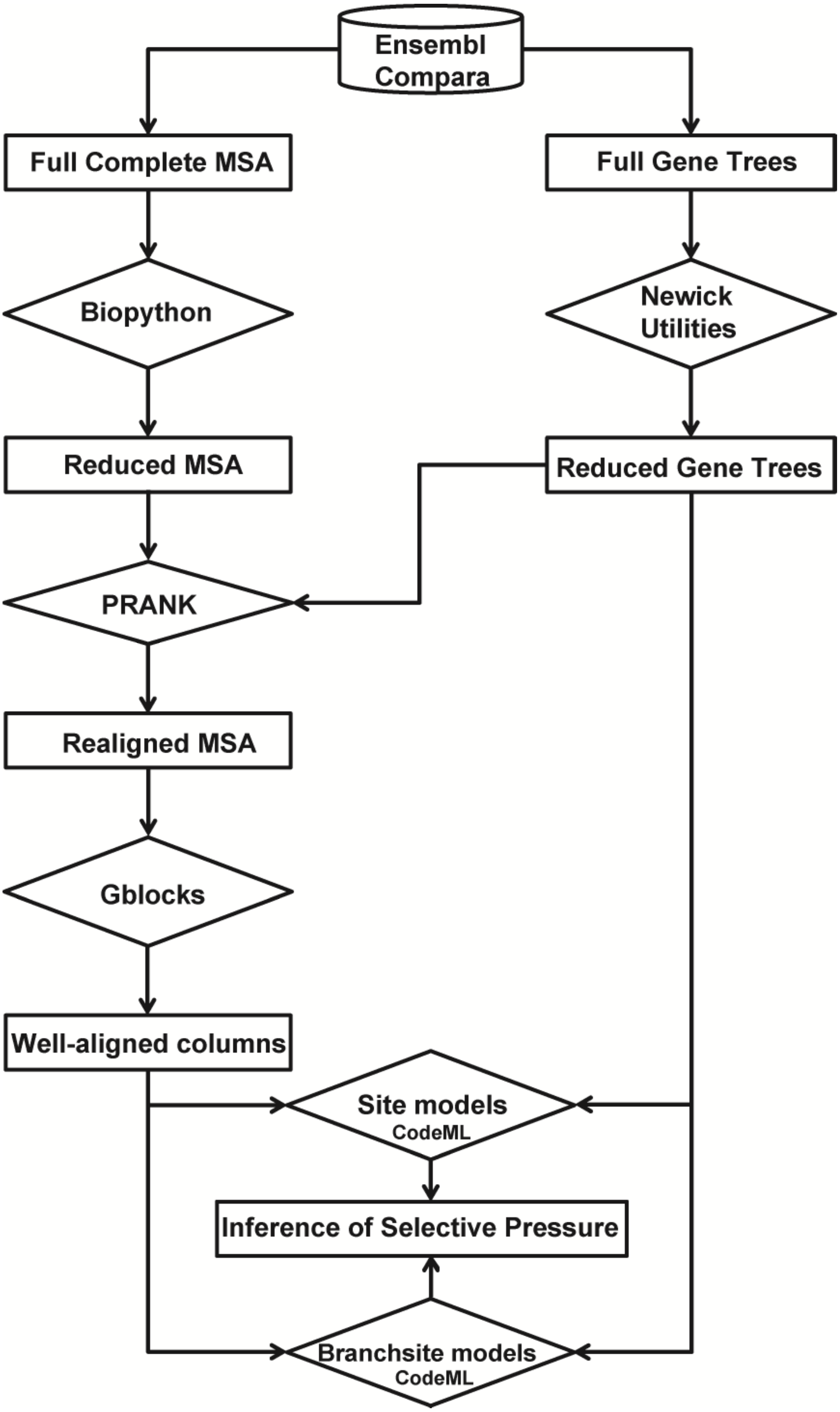
Ensembl Compara
Full Complete MSA
Full Gene Trees
Biopython
Newick Utilities
Reduced MSA
Reduced Gene Trees
PRANK
Realigned MSA
Gblocks
Well-aligned columns
Site models
CodeML
Inference of Selective Pressure
Branchsite models
CodeML

FIGURE S1

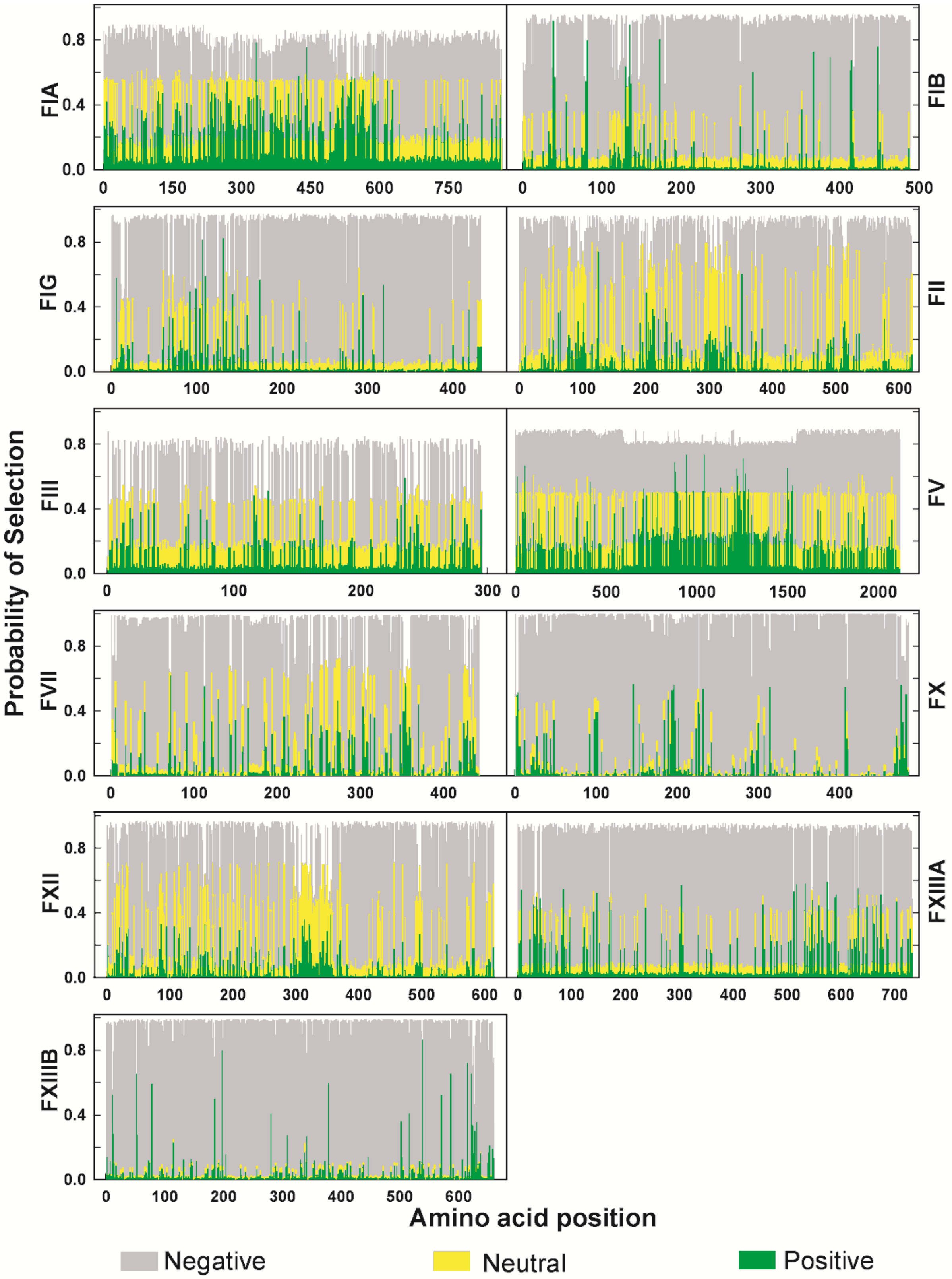

Probability of Selection
Amino acid position
FIA
FIB
FIG
FII
FIII
FV
FVII
FX
FXII
FXIIIA
FXIIIB
Negative
Neutral
Positive

FIGURE S2
